# New aspects of endocrine control of atrial fibrillation and possibilities for clinical translation

**DOI:** 10.1093/cvr/cvab080

**Published:** 2021-03-16

**Authors:** Martin Aguilar, Robert A Rose, Abhijit Takawale, Stanley Nattel, Svetlana Reilly

**Affiliations:** 1 Department of Medicine and Research Center, Montreal Heart Institute and Université de Montréal, Montréal, QC, Canada; 2 Department of Pharmacology and Physiology/Institute of Biomedical Engineering, Université de Montréal, Montréal, QC, Canada; 3 Department of Cardiac Sciences, Department of Physiology and Pharmacology, Libin Cardiovascular Institute, Cumming School of Medicine, Health Research Innovation Center, University of Calgary, AB, Canada; 4 Department of Pharmacology and Therapeutics, McGill University, Montreal, QC, Canada; 5 Faculty of Medicine, Department of Pharmacology and Physiology, and Research Centre, Montreal Heart Institute and University of Montreal, Montreal, QC, Canada; 6 Institute of Pharmacology, West German Heart and Vascular Center, Faculty of Medicine, University Duisburg-Essen, Germany; 7 IHU LIRYC and Fondation Bordeaux Université, Bordeaux, France; 8 Division of Cardiovascular Medicine, Radcliffe Department of Medicine, British Heart Foundation Centre of Research Excellence, University of Oxford, John Radcliffe Hospital, Oxford, UK

**Keywords:** Atrial fibrillation, Arrhythmia, Endocrine system, Heart

## Abstract

Hormones are potent endo-, para-, and autocrine endogenous regulators of the function of multiple organs, including the heart. Endocrine dysfunction promotes a number of cardiovascular diseases, including atrial fibrillation (AF). While the heart is a target for endocrine regulation, it is also an active endocrine organ itself, secreting a number of important bioactive hormones that convey significant endocrine effects, but also through para-/autocrine actions, actively participate in cardiac self-regulation. The hormones regulating heart-function work in concert to support myocardial performance. AF is a serious clinical problem associated with increased morbidity and mortality, mainly due to stroke and heart failure. Current therapies for AF remain inadequate. AF is characterized by altered atrial function and structure, including electrical and profibrotic remodelling in the atria and ventricles, which facilitates AF progression and hampers its treatment. Although features of this remodelling are well-established and its mechanisms are partly understood, important pathways pertinent to AF arrhythmogenesis are still unidentified. The discovery of these missing pathways has the potential to lead to therapeutic breakthroughs. Endocrine dysfunction is well-recognized to lead to AF. In this review, we discuss endocrine and cardiocrine signalling systems that directly, or as a consequence of an underlying cardiac pathology, contribute to AF pathogenesis. More specifically, we consider the roles of products from the hypothalamic-pituitary axis, the adrenal glands, adipose tissue, the renin–angiotensin system, atrial cardiomyocytes, and the thyroid gland in controlling atrial electrical and structural properties. The influence of endocrine/paracrine dysfunction on AF risk and mechanisms is evaluated and discussed. We focus on the most recent findings and reflect on the potential of translating them into clinical application.

## 1. The hypothalamus-pituitary axis and AF

**Figure 1:**
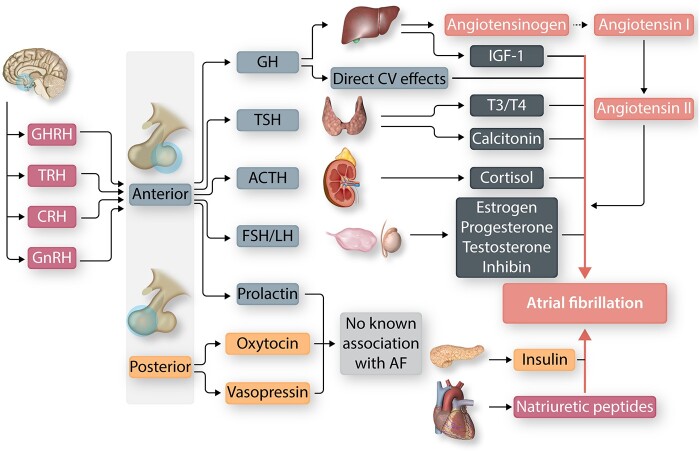
Summary of the endocrine glands and hormones associated with atrial fibrillation (AF). The anterior pituitary secretes tropic hormones in response to hypothalamic stimulation. Tropic hormones stimulate the release of physiologically active hormones from their target organ(s). The posterior pituitary hormones do not have known direct electrophysiological effects. ACTH, adrenocorticotropic hormone; CRH, corticotropin-releasing hormone; CV, cardiovascular; FSH, follicle-stimulating hormone; GH, growth hormone; GHRH, GH releasing-hormone; GnRH, gonadotropin-releasing hormone; IGF-1, insulin-like growth factor-I; LH, luteinizing hormone; T3, triiodothyronine; T4, tetraiodothyronine; TRH, thyrotropin-releasing hormone.

The hypothalamus-pituitary axis is a key component of the endocrine system. The pituitary gland is located in the sella turcica, a pouch of the sphenoid bone, and connected to the hypothalamus via the hypophyseal stalk. It is composed of the anterior (adenohypophysis) and posterior (neurohypophysis) pituitary, embryologically separate, and functionally independent units. The posterior pituitary secretes oxytocin and vasopressin synthesized in the hypothalamus; they do not have any known direct electrophysiological effects or associations with atrial fibrillation (AF). The anterior pituitary secretes the six tropic hormones, thyroid-stimulating hormone (TSH), adrenocorticotropic hormone (ACTH), growth hormone (GH), follicle-stimulating hormone (FSH), luteinizing hormone (LH), and prolactin, in response to stimulation from the hypothalamus. Over or underproduction of pituitary hormones lead to characteristic disease-conditions (*Figure 1*).

ACTH stimulates cortisol production by the adrenal cortex. Cushing’s disease is caused by pituitary ACTH-dependent hypercortisolism.^1^ Cushing’s syndrome can be caused by ectopic ACTH-secreting extra-pituitary tumours and iatrogenic glucocorticoid administration. AF is more prevalent in patients taking high-dose exogenous corticosteroids^2^ and in those with cortisol-secreting adrenal adenomas vs. age-matched controls^3^; the association between Cushing’s disease *per se* and AF is not well-characterized and the underlying mechanisms are incompletely understood. Cushing’s syndrome/disease causes important AF-associated risk factors, like hypertension, diabetes, dyslipidaemia, and obesity.^4^^,^^5^ Adrenal insufficiency results in hypocortisolism and can be primary (adrenal), secondary (pituitary), or tertiary (hypothalamus). Association between adrenal dysfunction and AF has not been described.

Glucocorticoid receptors (GRs) are expressed in cardiomyocytes and mediate a wide range of genomic and non-genomic anabolic and metabolic effects. The electrophysiological effects of GR activation, particularly with respect to AF,^6^ are understudied; however, cortisol affects intracellular calcium (Ca^2+^) homeostasis. Hydrocortisone administration led to a protein kinase C (PKC)-dependent shortening of the ventricular action potential duration (APD) and changes in Ca^2+^-transients^7^, while adrenalectomized rats showed abnormal sarcoplasmic Ca^2+^-uptake correctable with exogenous dexamethasone.^8^ These effects may be due to serum- and glucocorticoid-inducible kinase 1 (SGK1)-mediated upregulation of cardiac potassium (K^+^) currents, including I_to_, I_Ks_, I_Kr_, and I_Kur_.^9^^,^^10^ Adrenal insufficiency is associated with QT-prolongation and polymorphic ventricular tachycardia responsive to exogenous corticosteroids.^11^^,^^12^ While excess cortisol increases the risk of AF, likely mediated by a combination of direct electrophysiological effects and indirectly through AF-associated risk factors, the effects of cortisol deficiency on atrial electrophysiology and AF are unknown. Recent studies have identified the importance of GR-transcriptionally induced glucocorticoid-induced leucine zipper (GILZ or tsc22d3) protein. GILZ mediates multiple effects of glucocorticoids that has not been linked to AF potentially due to more selective, as opposed to glucocorticoids, effects.^13^

**Figure 2:**
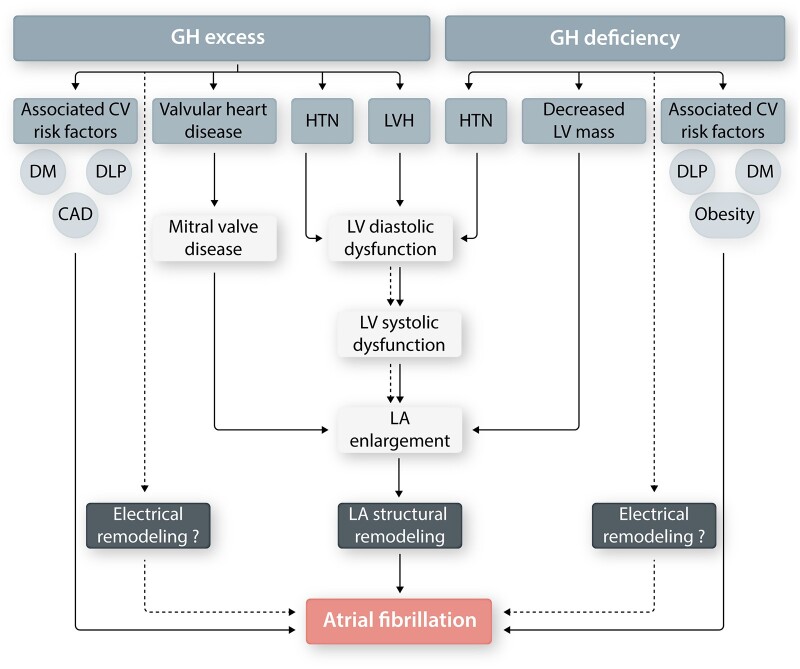
Growth hormone (GH) and AF. GH excess and deficiency have both been associated with an increased risk of AF. Chronic GH excess leads to left-ventricular (LV) diastolic dysfunction and left-atrial (LA) enlargement, contributing to pro-arrhythmic LA structural remodelling. In addition, GH deficiency has been associated with decreased LV mass and LV systolic dysfunction. GH dysregulation also often co-exists with pro-AF cardiovascular (CV) risk factors [i.e. hypertension (HTN), diabetes (DM), dyslipidaemia (DLP), coronary artery disease (CAD)]. The effect of GH excess and/or deficiency, if any, on LA electrophysiology (electrical remodelling) is not known.

GH is secreted by the anterior pituitary and has direct as well as indirect anabolic and positive inotropic effects mediated via the GH-directed hepatic secretion of insulin-like growth factor (IGF)-1.^14^ Both GH excess and deficiency have been associated with increased cardiac arrhythmias, including AF, cardiovascular morbidity, and mortality.^15^ Chronic GH excess, most often caused by a pituitary GH-secreting adenoma, leads to acromegaly, with typical morphological and clinical features. Elevated GH is associated with left ventricular (LV) hypertrophy, left-sided valvular heart disease, and other cardiovascular risk factors for AF, including diabetes, coronary artery disease, hypertension, and dyslipidaemia.^16^ The mechanism underlying acromegaly-associated AF has not been fully elucidated but likely involves left atrial (LA) enlargement with pro-fibrillatory structural remodelling (*Figure 2*).

GH deficiency, a common manifestation of hypopituitarism, presents with growth retardation in children and as a cardiometabolic syndrome in adults.^15^ Patients with GH deficiency GH develop hypertension, reduced LV mass (*Figure 2*),^17^ and LA structural remodelling that may mediate increased AF risk in patients with GH deficiency. Cardiomyocytes express GH and IGF-1 receptors but there are no reported direct effects of GH/IGF-1 on atrial electrophysiology.^18^

FSH and LH stimulate oestrogen production in women (ovaries) and testosterone in men (testis), among other functions. Changes in cardiac electrophysiology across the menstrual cycle have been described, with oestrogen having APD-prolonging effects (follicular phase) while progesterone tends to shorten APD (luteal phase) by decreasing and increasing K^+^ repolarizing currents, respectively^19^; however, oestrogen abnormalities have not been formally linked to a higher risk of AF.

Low testosterone levels can result from primary (testis), secondary (pituitary), or tertiary (hypothalamus) male hypogonadism in young individuals, or as the result of normal ageing in older men.^20^ Males with lower testosterone levels have worse cardiovascular outcomes in general, including higher rates of AF.^21^ Low testosterone levels are associated with several AF-related cardiovascular risk factors such as diabetes, premature coronary artery disease, dyslipidaemia, and obesity. Accordingly, recent work suggests that transgender women on hormonal therapy might be at increased risk of AF^22^; however, the available data are scarce, and a larger database is needed to confirm this association.

Furthermore, proinflammatory markers like C-reactive protein (CRP) and interleukin-6 (IL-6) have an inverse correlation with testosterone levels,^23^ suggesting that inflammation may contribute to pro-fibrillatory atrial structural remodelling. Testosterone affects cardiac electrophysiology, as castrated rats showed increased expression of the ryanodine receptor type-2 (RyR2), sodium (Na^+^)/Ca^2+^ exchanger (NCX), late Na^+^ current (I_NaL_), APD prolongation, and higher AF susceptibility vs. control animals.^24^^,^^25^ Androgen receptor knock-out (KO) rats have reduced resting membrane potential, increased APD, and enhanced sensitivity to isoproterenol-induced delayed afterdepolarizations,^26^ that are partly reversed by testosterone replacement therapy.^20^

A TSH-producing adenoma is the most common cause of central hyperthyroidism, a rare cause of thyrotoxicosis. Similarly, central hypothyroidism is much less common than its primary counterpart. Although poorly characterized, the cardiovascular consequences of central hypo-/hyperthyroidism are expected to parallel those observed with primary hypo-/hyperthyroidism, as described below.

Prolactin stimulates milk production in the gravid woman and has not been association with AF.

## 2. The adrenals and AF

The adrenal, or suprarenal, glands are composed of two embryologically distinct and functionally independent cortical and medullary units. The adrenal cortex contains three layers secreting aldosterone (zona glomerulosa), cortisol (zona fasciculata), and androgens (zona reticularis). The adrenal medulla is part of the sympathetic nervous system and releases epinephrine and norepinephrine.

Aldosterone is normally secreted in response to hypovolemia and hyperkalaemia and binds mineralocorticoid receptors (MRs) to regulate Na^+^/K^+^/H^+^ homeostasis by activating the epithelial Na^+^ channel (ENaC) in the nephron distal tubule and collecting duct.^27^ MRs are also expressed on other cell types, including atrial and ventricular cardiomyocytes. The association between aldosterone and AF has most clearly been demonstrated in patients with primary hyperaldosteronism (PA; renin-independent aldosterone production), the most common cause of secondary hypertension.^28^ AF is more prevalent in patients with PA (7.3%) vs. matched patients with essential hypertension (0.6%)^29^, adrenalectomy lowers the PA-associated risk of AF^30^ and the MR-specific antagonist eplerenone is associated with a 42% relative risk reduction of AF,^31^ all suggesting a potential role of aldosterone in the pathogenesis of AF independent of upstream regulatory hormones (i.e. renin/angiotensin-II).

**Figure 3:**
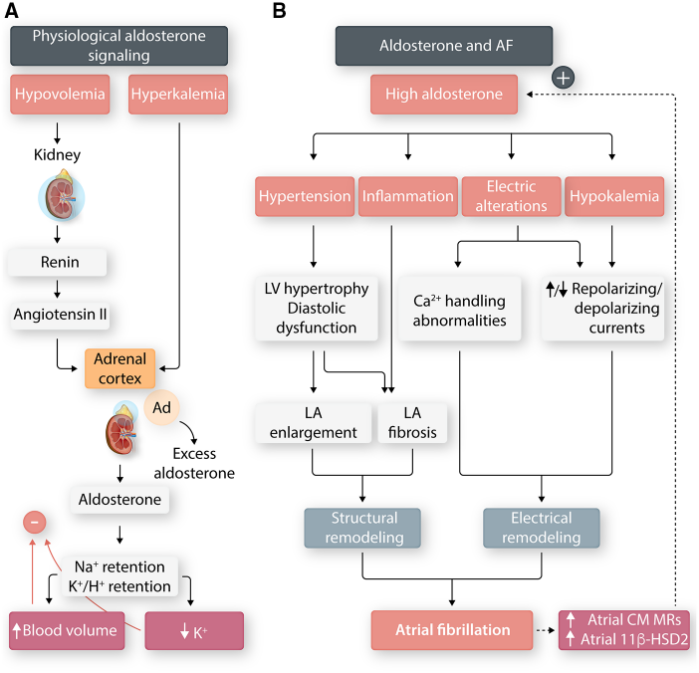
Hyperaldosteronism and AF. (*A*) Hypovolemia and hyperkalaemia are the primary physiological stimuli for adrenal aldosterone secretion, which acts on the nephron distal tubule and collecting duct to retain Na^+^ and excrete K^+^. (*B*) Mechanism of aldosterone-related AF. Hyperaldosteronism causes angiotensin-independent hypertension and left atrial (LA) inflammation, leading to pro-fibrillatory LA remodelling. It also produces pro-AF electrical remodelling in the form of LA action potential-shortening, increased sarcoplasmic reticulum Ca^2+^ sparks, and delayed afterdepolarizations. Sustained AF may potentiate the effects of hyperaldosteronism by upregulating of the mineralocorticoid receptor (MR) on AF atrial cardiomyocytes (CMs). Furthermore, AF increases 11b-hydroxysteroid dehydrogenase type 2 (11b-HSD2), which metabolizes cortisol, thereby increasing MR occupancy by aldosterone.

The mechanisms linking hyperaldosteronism and AF are complex and incompletely understood (*Figure 3*). However, chronic excess aldosterone leads to LA enlargement and remodelling, indirectly via hypertension and diastolic dysfunction and/or directly via blood pressure-independent effects.^32–34^ Aldosterone-binding to cardiac macrophage MRs increases expression of the profibrotic markers transforming growth factor-beta 1 (TGF-β1), matrix metalloproteinase-12, tumour necrosis factor-alpha (TNFα), and plasminogen activator inhibitor-1 (PAI-1)^35^^,^^36^, promotes macrophage-mediated oxidative stress^37^ and stimulates the Keep1/Nrf2-dependent cardiac fibroblast to myofibroblast transformation^38^ contributing to pro-fibrillatory LA structural remodelling. Conduction time and P-wave duration are increased in a rat model of hyperaldosteronism, compatible with increased atrial fibrosis and slowed conduction.^39^

Aldosterone also has direct electrophysiological effects, the clinical significance of which remains to be fully elucidated. Aldosterone administration to rats caused APD shortening, mediated by an increase in Kir2.1 (inward rectifier K^+^ current, I_K1_) and Kv1.5 (ultrarapid delayed rectifier K^+^ current, I_Kur_) expression; these changes were reversed by the MR antagonist spironolactone.^40^ Conversely, ventricular APD was prolonged in a MR-overexpression model because of the downregulation of transient outward K^+^ current (I_to_) and upregulation of L-type Ca^2+^ current (I_CaL_).^41^ It has been shown that ventricular I_CaL_ magnitude correlates with aldosterone levels^42^^,^^43^ and MR-activation increases sarcoplasmic-reticulum (SR) Ca^2+^-sparks and delayed afterdepolarizations,^44^ implicated in AF pathophysiology. A mouse model of spontaneous AF was associated with increased 11β-hydroxysteroid dehydrogenase type 2 (11β-HSD2), which inactivates cortisol, thereby allowing for increased MR occupancy by aldosterone.^45^ Patients with AF have upregulated expression of MRs vs. sinus-rhythm controls^46^ and MR antagonists reduce the risk of AF in heart failure patients.^31^ Hence, AF may itself potentiate aldosterone’s proarrhythmic effects.

Pheochromocytomas are epinephrine and norepinephrine-secreting adrenal tumours. The excess catecholaminergic state leads to hypertension, myocardial ischaemia/or, and cardiomyopathy.^47^ Cardiac arrhythmias are common in pheochromocytoma and AF was documented in 11.3% of patients with this condition, all of which responded to tumour resection.^48^

## 3. Obesity and AF

**Figure 4:**
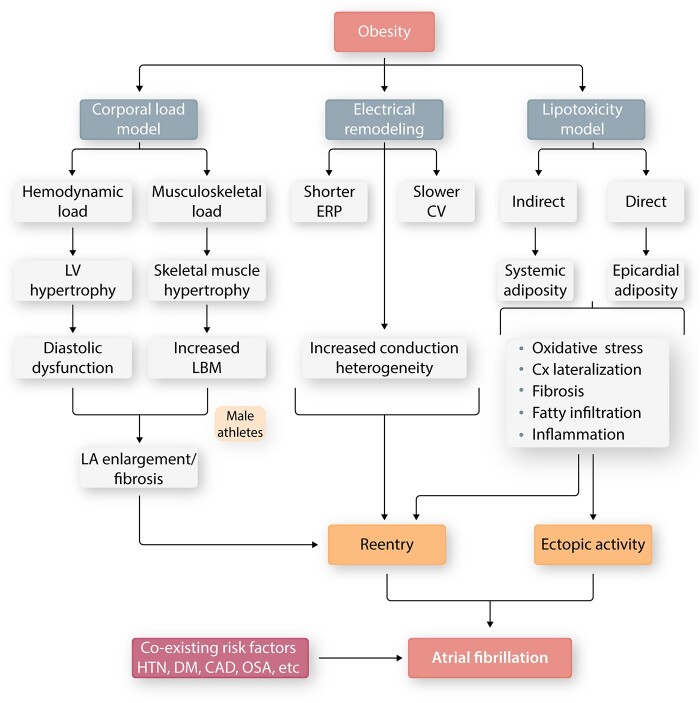
Current understanding of the mechanistic links between obesity and AF. The corporal load model states that excess body mass (adipose and/or lean) poses a haemodynamic load culminating in pro-AF left-atrial (LA) remodelling. Obesity has also been associated with pro-fibrillatory electrical remodelling in the form of shorter effective refractory period (ERP), slower conduction velocity (CV) and increased conduction heterogeneity. Adipocytes have a pro-inflammatory secretome which can affect LA electrophysiology indirectly (systemic adiposity) or directly (epicardial adiposity). Finally, obesity often co-exists with a number of pro-AF cardiovascular risk factors.

Obesity and AF are both reaching epidemic levels. Obesity can result from endocrine abnormalities, and adipose tissue secretes a number of bioactive hormones.^49^ Obesity is a well-established AF risk factor.^45^ Weight loss reduces the risk of AF in obese patients improves outcomes after AF catheter ablation and reverses obesity-related electrical remodelling.^50^ The relationship between obesity and AF has been framed into a two-component model: the *corporal load model* and the *lipotoxicity model* (*Figure 4*).^51^

The corporal load model stipulates that the increase in hemodynamic load imposed by excess weight leads to left ventricular (LV) hypertrophy, diastolic dysfunction with secondary LA enlargement, and pro-fibrillating remodelling. Interestingly, excess lean body mass, not merely adipose mass, is an important mediator of obesity-associated AF risk.^52^

The lipotoxicity model refers to the direct proinflammatory and profibrotic states associated with obesity. Obesity induces pro-inflammatory signalling in the atria^53^ that can promote both ectopic firing and an AF-maintaining electrical and structural substrate, ultimately leading to AF.^54^ Visceral adiposity is associated with increased blood leukocyte count, CRP, IL-6, and TNF-α.^55^^,^^56^ Accelerated fibrogenesis mediated by the TGF-β1, connective tissue growth factor (CTGF), and endothelin-1 systems,^57^ among others, contributes to the proarrhythmic lipotoxic effect. Epicardial adipose tissue (EpAT) is in direct contact with the epicardium and shares its blood supply, positioning it to affect atrial electrophysiology through paracrine and vasocrine interactions. EpAT volume correlates with areas of atrial fibrosis, slow conduction, electrogram fractionation, and lateralization of connexin (Cx)-40,^58^ suggesting a direct effect on atrial electrophysiology. Similar to visceral fat, EpAT secretes metabolically active (e.g. free fatty acids), angiogenic (e.g. vascular endothelial growth factor), growth (e.g. activin A), and remodelling (TGF-β1/2 and MMPs) factors, as well as inflammatory cyto- and chemokines (e.g. IL-6 and PAI-1), and adipokines (e.g. leptin).^59^ While leptin is implicated in angiotensin-II-mediated pro-fibrillatory atrial remodelling,^60^^,^^61^ another adipokine, resistin, correlates with clinical AF risk.^62^ Adipocytes also secrete neprilysin, a neutral endopeptidase that degrades cardioprotective endogenous natriuretic peptides (NPs),^63^ that positively correlates with body mass index (BMI)^64^ and regulates angiotensin-II concentrations in human adipose tissue.^65^ An overproduction of aldosterone,^66^ linked to AF pathogenesis, shorter atrial, and pulmonary vein refractory periods, conduction slowing, and heterogeneity^57^^,^^67^ are also found obesity and may be involved in obesity-associated AF.

## 4. Renin–angiotensin system and AF

Hypertension, an important risk factor for AF,^68–71^ is often associated with activation of the renin–angiotensin system (RAS).^72^ The RAS system is a neuroendocrine axis involving kidney production of renin that converts liver-produced angiotensin into angiotensin-I, which is subsequently converted into active circulating angiotensin-II in the lungs. Hypertension leads to atrial remodelling as indicated by LA enlargement and prolongation of P-wave duration.^73^^,^^74^

**Figure 5:**
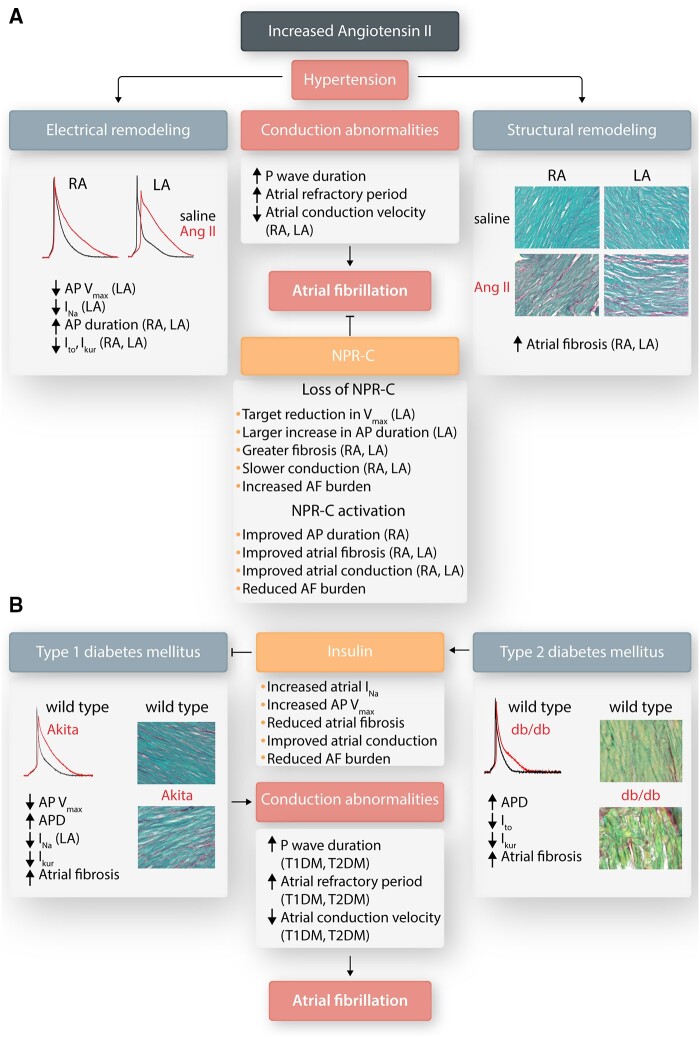
Atrial electrical and structural remodelling in angiotensin-II mediated hypertension and in mouse models of type-1 (T1DM) and type-2 (T2DM) diabetes mellitus. (*A*) Angiotensin-II infusion in mice causes distinct patterns of ion channel remodelling and changes in action potential morphology in left and right atrial myocytes. Angiotensin-II also causes right and LA fibrosis. These alterations lead to conduction abnormalities and increased susceptibility to AF. Loss of NPR-C leads to worsened ion channel remodelling and atrial fibrosis, as well as enhanced AF susceptibility, while NPR-C activation prevents some ion channel remodelling, reduces right and LA fibrosis, and decreases AF burden. (*B*) T1DM (Akita mice) is associated with reductions in AP V_max_ due to reduction in atrial I_Na_ as well as increases in AP duration due to reduction in I_Kur_. T2DM (db/db mice) show increases in AP duration due to reduction in both I_to_ and I_Kur_ while I_Na_ amplitude and AP V_max_ are not altered. Both T1DM and T2DM are associated with increased atrial fibrosis. These alterations lead to conduction abnormalities and increased susceptibility to AF. Insulin treatment in T1DM prevents reductions in atrial I_Na_ and reduces atrial fibrosis leading to improved conduction and reduced AF susceptibility.

Angiotensin-II infusion produces a rapid increase in systolic blood pressure (exceeding 140–150 mmHg)^75–77^ and a substantial increase in susceptibility to AF in mice.^75–79^ Enhanced AF-susceptibility in angiotensin-II infused mice occurs in association with atrial enlargement, atrial fibrosis, and prolonged P-wave duration.^75^^,^^80^^,^^81^ Consistent with P-wave prolongation *in vivo*, optical mapping demonstrates conduction slowing in the right atrium (RA) and LA of angiotensin-II infused mice.^75^ RA and LA APD are prolonged and sinoatrial node function is impaired in angiotensin-II infused mice (*Figure 5A*).^75^ Notably, atrial tachyarrhythmia itself induces angiotensin-II type 1 receptor-mediated oxidative stress (mainly due to increased nicotinamide adenine dinucleotide phosphate oxidase activity, LOX-1 upregulation, and F_2_-isoprostane generation) in the ventricular myocardium, negatively impacting on its function.^82^ Ion-channel remodelling may explain the electrophysiological changes associated with AF promotion by angiotensin-II. LA I_Na_ is reduced by approximately 50% in angiotensin-II infused mice, apparently via enhanced PKCα activity as dialysis with BIM1 (a PKC inhibitor) normalized I_Na_ density and activation kinetics.^69^^,^^71^ APD-prolongation occurred in conjunction with decreased outward K^+^-current (I_K_), attributed to reductions in I_to_ and I_Kur_ independently of a change in K_v_4.2, K_V_4.3, KChIP2, and K_v_1.5 protein levels.

AF following angiotensin-II infusion in mice is also associated with oxidative stress, leading to oxidation of Ca^2+^-calmodulin-dependent protein kinase II (CaMKII).^78^ CaMKII-oxidation leads to pathological, constitutively active CaMKII-signalling.^83^ Oxidized CaMKII expression is increased in both AF patients and mice infused with angiotensin-II.^78^ CaMKII oxidation causes arrhythmogenic alterations in SR Ca^2+^-handling, with increased Ca^2+^-sparks leading to delayed afterdepolarizations. Knock-in mice resistant to CaMKIIδ oxidation are protected from Ca^2+^-mishandling and show less AF inducibility.^78^

Angiotensin-II infusion also causes atrial interstitial fibrosis^75^^,^^77^ (*Figure 5A*), resulting from altered extracellular matrix (ECM) remodelling by MMPs and TIMPs under the influence of oxidative stress and inflammation.^78^^,^^79^

## 5. Natriuretic peptides in AF

Natriuretic peptides (NPs) are cardioprotective hormones that play important roles in regulating cardiac electrophysiology and arrhythmogenesis.^84^^,^^85^ NPs modulate atrial AP morphology and alter atrial conduction patterns by regulating ion-channel function.^84–88^ NPs elicit their effects by binding to NP receptors (NPRs), including NPR-A, NPR-B, and NPR-C.^89^ NPR-A and NPR-B are guanylyl cyclase-linked receptors that modulate cGMP signalling, while NPR-C is coupled to the inhibitory G protein (Gi) and phospholipase C signalling.^84^^,^^90^^,^^91^

NPR-C is highly expressed in the atria,^86^^,^^92^ and recent studies have identified an essential role for NPR-C in regulating atrial conduction and AF inducibility.^77^^,^^92^ NPR-C knockout (NPR-C^−/−^) mice display increased susceptibility to burst pacing-induced AF in association with impaired atrial conduction. Atrial interstitial fibrous-tissue content is increased in NPR-C^−/−^ mice, whereas no differences in atrial AP morphology occur.^92^ In contrast, no ventricular arrhythmias or ventricular fibrosis were observed in NPR-C^−/−^ mice,^92^ further indicating that NPR-C is particularly important for regulation of atrial structure and function.

NPR-C plays a modulating role in angiotensin-II mediated AF.^77^ Angiotensin-II infusion in NPR-C^−/−^ mice produces enhanced effects on AF vulnerability and duration, P-wave duration, and atrial conduction. Reductions in AP upstroke velocity (V_max_) and I_Na_, as well as APD prolongation, are seen, particularly in LA cardiomyocytes. Angiotensin-II infusion also produced larger increases in PKCα protein expression in NPR-C^-/-^ mice, as well as enhanced RA and LA fibrosis.^77^ Co-treatment of wild-type mice with angiotensin-II and cANF (a synthetic, selective NPR-C agonist^90^) reduces AF burden, while improving atrial conduction, attenuating atrial fibrosis, and improving AP properties.^77^ These findings are consistent with other work showing that NPR-C plays protective roles in the cardiovascular system.^76^^,^^93–95^ On the other hand, one study using transverse aortic constriction and TGF-β-overexpression found that the absence of NPR-C was protective against AF and atrial fibrosis.^96^ The basis for these contradictory observations is unclear and further studies are warranted.

Studies showed that mutations in the atrial natriuretic peptide (ANP) gene are linked to AF.^97^^,^^98^ A family with familial AF was shown to have a frameshift mutation in the *NPPA* gene (encoding ANP) that results in a mutated ANP (mANP) circulating at concentrations 5–10 times greater than wild-type ANP because of increased resistance to proteolytic degradation.^98^^,^^99^ While ANP has been shown to increase funny current (*I*_f_) in human atrial cardiomyocytes and predispose to AF,^100^ ANP and mANP have also been found to demonstrate opposite effects on mouse and human atrial cardiomyocytes.^101^ Specifically, ANP increased V_max_, APD, and I_Ca,L_ in isolated atrial cardiomyocytes via the NPR-A receptor. In intact mouse atrial preparations, ANP speeded atrial conduction and increased atrial effective refractory period (AERP). In contrast, mANP decreased atrial V_max_, shortened atrial APD, decreased atrial I_Ca,L_, slowed atrial conduction, and shortened AERP. These effects were mediated by the NPR-C receptor, as the effects of mANP were absent in NPR-C^−/−^ mice. ANP and mANP also had opposing effects on I_Ca,L_ in human RA cardiomyocytes. Finally, mANP administration caused re-entrant conduction patterns, ectopic firing, and disorganized conduction in mouse atria exposed to programmed stimulation, an effect not seen with ANP. These studies suggest that mANP is proarrhythmic in association with a shift in the balance between NPR-A and NPR-C mediated NP-signalling. Mice expressing the same *nppa* frameshift mutation show increased AF burden in association with APD shortening, in association with ion-channel remodelling, including changes in amplitude and expression of Na^+^, Ca^2+^, and K^+^ channels.^94^ Collectively, the available data indicate that this frameshift *nppa* mutation increases susceptibility to AF in association with increased circulating mANP levels and shortening of the atrial AP, which could decrease the wavelength for re-entry (*Figure 5A*).

## 6. AF in diabetes mellitus

Type-1 (T1DM) and type-2 (T2DM) diabetes mellitus (DM) are metabolic disorders associated with hyperglycaemia and changes in insulin production and signalling.^102^^−^^104^ T1DM is characterized by the loss of insulin-producing β-islet cells in the pancreas and deficient insulin generation. T2DM, which often occurs in association with obesity, is characterized by insulin resistance in peripheral tissues while insulin can still be produced in the pancreas.^102^ T2DM can ultimately lead to insulin insufficiency requiring insulin therapy.

AF is prevalent in both T1DM and T2DM.^69^^,^^71^^,^^105^ T1DM is associated with atrial electrical and structural remodelling.^69^ Experimental studies have evaluated atrial effects in genetic (Akita mice) or chemically induced [streptozotocin (STZ) or alloxan] animal models, of T1DM^106^ that are characterized by substantial increases in AF susceptibility and duration.^107^^,^^108^ Akita and STZ mice had increased P-wave duration; Akita mice also had impaired RA and LA conduction,^107^ reduced atrial V_max_, and prolonged APD. These AP morphology changes occurred in association with reductions in I_Kur_ and I_Na_, associated with decreased *SCN5a* gene and Na_V_1.5 protein expression, as well as a loss of phosphoinositide 3-kinase (PI3K) signalling via the second messenger PIP_3_. Strikingly, insulin treatment protected against changes in I_Na_, but not the changes in I_Kur_, in Akita mice. Chronic insulin treatment increased Na_V_1.5 protein levels, atrial I_Na_ density, and AP upstroke velocity. Insulin could also increase atrial I_Na_ and AP upstroke velocity acutely via the rapid activation of PIP_3_ signalling. These effects of insulin on atrial I_Na_ were associated with increases in atrial conduction velocity and were sufficient to reduce the AF burden. Consistent with previous work on the PI3K- and PIP_3_-mediated effects on Na^+^-channel function,^109^ these studies revealed a critical role for insulin in regulating atrial electrophysiology and AF susceptibility via effects on atrial Na^+^ channels in T1DM, though the basis for I_Na_ dysregulation in T1DM remains unclear. Structural profibrotic remodelling in T1DM^110^^−^^112^ is increased in the atria of Akita mice,^110^ STZ-treated rodents,^111^^−^^113^ and alloxan-treated rabbits,^114^ that in STZ-treated rats was prevented by inhibition of the type-1 angiotensin-II receptors,^112^ suggesting a critical role of angiotensin-II in T1DM-related atrial fibrosis (*Figure 5B*). STZ-treated rats showed reduced velocity and increased heterogeneity of the LA conduction associated with a reduced LA Cx40 expression, leading to increased arrhythmogenesis,^113^ while atrial Cx40 or Cx43 mRNA expression were unaltered in Akita mice.^107^

T2DM, which accounts for up to 90% of DM-patients, continues to increase at epidemic proportions in association with rising rates of obesity and metabolic syndrome.^115^ AF is prevalent in T2DM.^105^^,^^116^ Clinical studies in T2DM patients have shown that alterations in Ca^2+^-handling may contribute to atrial remodelling.^117^ T2DM patients have atrial interstitial fibrosis and increased EpAT, potentially infiltrating atrial myocardium,^118^^−^^123^ that could lead to impaired electrical conduction and AF (*Figure 5B*). While mechanistic studies in animal models are limited, recent work in T1DM and T2DM mouse models demonstrated that AF promotion is related to pro-arrhythmic activation of CaMKII (due to oxidative stress-mediated oxidation and O-GlcNAcylation of CaMKII)^124^ that would potentially affect multiple ion currents.^83^

AF in T2DM was recently investigated in db/db mice, which carry a mutation in the leptin receptor leading to obesity, insulin resistance, and hyperglycaemia.^125^ The db/db mice have increased susceptibility to burst-pacing induced AF, associated with increased P-wave duration and AERP, reduced RA and LA conduction velocity, and prolonged heterogeneous APD. These changes were accompanied by a decrease in I_to_ (associated with reduced expression of *Kcnd2* mRNA and K_v_4.2 protein) and suppressed I_Kur,_ occurring in the presence of unchanged K_V_1.5 expression. Zucker diabetic fatty (ZDF) rats also showed increased AF susceptibility and APD,^126^ associated with reduced atrial I_to_, I_Kur_, and I_Ca,L_ currents, and respective channel protein subunits Kv4.3, Kv1.5, and Cav1.2, indicating some model-specific differences. In contrast to Akita (T1DM) mice,^107^ atrial I_Na_ was not reduced in db/db atrial myocytes.^125^ The only alteration observed in atrial I_Na_ in db/db mice was a shift in steady-state inactivation that resulted in a larger I_Na_ window current, which could contribute to the prolongation of APD. This observation identifies potentially important differences in electrical remodelling between T1DM and T2DM that may have important implications for AF therapy in these related, but distinct conditions.

Animal models of T2DM also consistently display atrial structural remodelling, including fibrosis, lipidosis, and inflammation (*Figure 5B*),^125^^,^^127–130^ which has been shown to promote AF.^54^ Adipokines (cytokines with pro-inflammatory properties) like leptin are also implicated in the atrial fibrosis of diabetic mice,^131^ while cathepsin-A (a proteolytic enzyme active in the extracellular space) contributes to atrial fibrosis in ZDF rats.^127^ Gene expression of Cx40 and Cx43 remained unchanged in both db/db mice and ZDF rats,^125^^,^^127^ yet lateralization of Cx43 was observed in ZDF rats^127^ that could underlie higher conduction heterogeneity.

## 7. Thyroid dysfunction in AF

Thyroid disease has a large number of well-characterized cardiac manifestations and both hypo- and hyperthyroidism have been associated with worse cardiovascular outcomes.^132^ Although clinical (low TSH, elevated T_4_) and subclinical (normal TSH, elevated T_4_) hyperthyroidism, even with marginally increased T_4_ levels, have been linked to a higher risk of AF,^133–135^ overt hyperthyroidism is present in less than 1% of patients with new-onset AF.^136^ Conversely, antiarrhythmic treatment with amiodarone is itself an important potential cause of hyperthyroidism in cardiac patients, so-called amiodarone-induced thyrotoxicosis.^137^ Elevated thyroid hormone (TH) levels have also been associated with increased atrial premature depolarizations and supraventricular tachyarrhythmias.^138^^,^^139^ Hypothyroidism appears to have relatively protective effects, especially against malignant ventricular arrhythmias, and a much less robust association with AF.^140^

**Figure 6:**
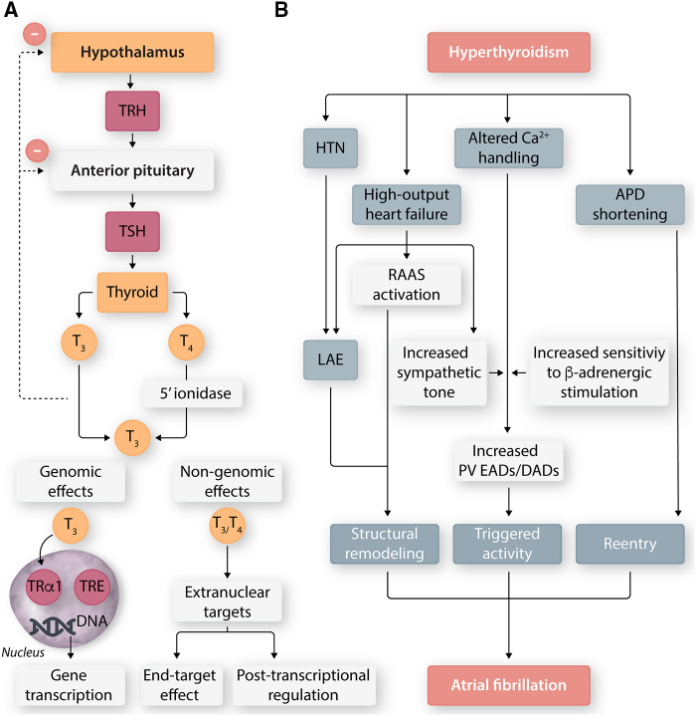
Thyroid dysfunction and AF. (*A*) The hypothalamic-pituitary-thyroid axis forms a closed negative-feedback system. The thyroid secretes primarily T4, which is converted to T_3_, the more metabolically active thyroid hormone, by the enzyme 5′-ionidase. Thyroid hormone effects can be genomic or non-genomic. Genomic effects are mediated by binding of T_3_ to the nuclear thyroid α-receptor-1 (TRα1), which interacts with the thyroid release elements (TREs) to promote/suppress thyroid hormone-regulated genes. Non-genomic effects are mediated by T_3_ and T_4_ as they interact with extra-nuclear receptors, which may or may not be structurally related to the thyroid receptor. (*B*) Hyperthyroidism leads to high-output heart failure (HF), causing left atrial enlargement (LAE), activation of the renin–angiotensin–aldosterone system (RAAS), and increased adrenergic stimulation. Altered intracellular Ca^2+^ promotes the formation of early (EADs) and delayed afterdepolarizations (DADs) from the pulmonary veins (PVs). Action potential duration (APD) shortening promotes re-entry. Finally, hypertension (HTN) also contributes to pro-fibrillatory left-atrial structural remodelling. TRH, thyrotropin-releasing hormone; TSH, thyroid-stimulating hormone; DNA, deoxyribonucleic acid.

Thyrotropin-releasing hormone (TRH) release from the hypothalamus stimulates secretion of TSH by the anterior-pituitary and subsequent release of tetraiodothyronine (thyroxine; T_4_) and in lesser amounts (∼1:9 ratio), triiodothyronine (T_3_) by the epithelial cells of the thyroid gland (*Figure 6*). The enzyme 5′-iodinase converts T_4_ into T_3_, the more metabolically active TH. T_3_ and T_4_ inhibit TRH and TSH release, forming a negative-feedback loop. TH actions can be genomic or non-genomic (*Figure 6*). Genomic effects are mediated as T_3_ enters the nucleus and interacts with the nuclear thyroid α receptor-1 (TRα1), which binds the thyroid release elements (TREs), promoting/repressing transcription of TH-regulated genes like sarcoplasmic reticulum Ca^2+^ adenosine triphosphate (SERCA2), phospholamban, Na^+^/K^+^ ATPase, NCX, selected voltage-gated K^+^ currents, I_CaL_, and β_1_-adrenergic receptor.^141^ Non-genomic effects (reviewed elsewhere^142^) have a rapid onset of actions that are transcription/translation-independent and are mediated by extra-nuclear receptors, structurally related to the thyroid receptor-like integrin αvβ3, cytoskeleton, mitogen-activated protein kinase ½ and PI3K.

Hyperthyroidism leads to so-called high-output heart failure characterized by hyperdynamic congestive failure in patients with normal baseline LV-function.^143^ Left untreated, heart failure leads to LA-enlargement, activation of the RAAS, and increased sympathetic tone (*Figure 6*)^142^ and hypertension.^142^

THs also have important electrophysiological effects. Hyperthyroidism leads to sinus tachycardia from increased rates of diastolic depolarization in sinoatrial cells; hypothyroidism has the opposite effect.^144^^,^^145^ Similarly, APD is consistently prolonged in animal models of hypothyroidism,^146–150^ whereas hyperthyroidism shortens APD.^147^^,^^148^^,^^151^ There are no documented effects of thyroid disease on the resting membrane potential. Beyond these well-characterized macroscopic electrophysiological changes, there is substantial heterogeneity in the reported TH-induced ion-channel remodelling.

### 7.1 Depolarizing currents

There are no documented effects of hypo-/hyperthyroidism on I_Na_, although one study reported increased/decreased conduction velocity in hyper-/hypothyroid rabbit atria, respectively.^146^ I_CaL_ has been found to be increased,^148^^,^^152^^,^^153^ unchanged^154^ or decreased^155^^,^^156^ in hyperthyroid models. Interestingly, one study found hyperthyroidism to increase I_CaL_ sensitivity to β-adrenergic stimulation, which may favour the occurrence of the triggered activity.^153^ Hence, the effects of hyperthyroidism on depolarizing currents are incompletely defined.

### 7.2 Repolarizing currents

THs were shown to increase I_K1_ by activating channel open probability, while the resting membrane potential remained unchanged.^146^^,^^148^ It was proposed that the rapid time course of action of T_3_ on I_K1_ is suggestive of a non-genomic mechanisms.^157^ THs have been reported to differentially increase ventricular I_to_ without affecting its atrial counterpart.^148^^,^^150^^,^^152^^,^^158^ Conversely, Kv1.4 was found to be reduced^159–161^ and Kv4.2 was unaffected^156^^,^^159–161^ by THs, while Kv1.5, the I_Kur_ α-subunit, was increased in hyperthyroid animals.^151^^,^^156^^,^^159–161^ APD prolongation due to decreased I_Ks_ was reported in thyroidectomized guinea pigs.^149^ Finally, Kv1.2 has been reported to be reduced^159^^,^^160^ and Kv2.1 unchanged^159^^,^^161^ or increased^151^^,^^160^ in hyperthyroid models. Hence, the mechanisms of hyperthyroidism-induced APD shortening are likely multifactorial.

THs also modulate intracellular Ca^2+^ homeostasis characterized by decreased phospholamban and increased SERCA2, potentially promoting the occurrence of proarrhythmic afterdepolarizations in hyperthyroid rats.^162^ Similarly, pulmonary vein cardiomyocytes from a hyperthyroid rabbits have higher automaticity, more frequent early and delayed afterdepolarizations and shorter APD.^163^ The pro-AF changes encountered in hyperthyroidism are summarized in *Figure 6*.

### 7.3 Thyroid dysfunction and cardiac remodelling

Dysregulated THs mediate multiple cardiac remodelling processes, e.g., necrosis, apoptosis, inflammation, and regression to the foetal heart phenotype. However, cardiac fibrosis remains the hallmark of AF-associated remodelling. THs were shown to downregulate interstitial collagen content^164^ and collagen I gene expression in the rat myocardium and cardiofibroblast culture^165^ and increased degradation of LV collagen-I/III protein, associated with an activation of MMPs and inhibition of TIMPs, in hyperthyroid rats.^166^^,^^167^ T3 supplementation in a rat model of ischemia/reperfusion inhibited TGF-β1, reduced scar size, and improved cardiac performance^168^; T3 was also shown to inhibit activator-protein-1 (AP-1),^169^ involved in the stimulation of MMPs and collagen mRNA.^170^ RAS appears to play a role in T4-induced cardiac hypertrophy,^171^ which was prevented by treatment with angiotensin-converting enzyme-inhibitor or angiotensin-1 receptor blocker.^172^ While hyperthyroidism in rats decreased LV levels of TGF-β1, SMAD2/3, total and phospho-serin368-Cx43 without enhanced interstitial collagen deposition, the TGF-β1 and SMAD2/3 were increased in hypothyroid rats.^173^ Although, the majority of studies argue for the anti-fibrotic effects of THs, longstanding hyperthyroidism has been demonstrated to impair LV function and increase interstitial fibrosis in hamsters.^174^ Thus, the TH-induced cardiac remodelling may continue to develop over time.

Hypothyroidism is primarily thought to exert profibrotic phenotype associated with increases in the LV TGF-β1 and procollagen-I mRNAs and protein,^164^ induced LV hypertrophy with fibrotic lesions, and upregulated α-SMA expression; all these changes were reversed by euthyroid state. Collagen-I/III gene expression was unaltered, while TGF-β1, CTGF, IL-1, and MCP1 gene expression were increased in hypothyroid rats.^175^ Interestingly, the TGF-β1 gene promoter has binding sites for Sp1, a critical transcription factor that interacts with TRH-binding protein associated factors,^176–178^ while pro-α1(I) collagen gene contains a binding site for the receptor, which functions as a TRE.^179^ Hypothyroidism was also shown to increase LV collagen-based diastolic wall stiffness^180^ and content of collagen and glycosaminoglycans in rat LV.^181^ Experiments in cultured rat cardiofibroblasts found increased biosynthesis of fibrillar collagen under TH-depleted conditions^179^ and increased proliferation in both TH-depleted^179^ and TH-treated cells.^165^

Atrial remodelling mediated by thyroid dysfunction is not fully understood; however, a recent study in rats showed that both hypo- and hyperthyroidism increased AF vulnerability, that, as well as the decreased LV and LA dimensions, AERP prolongation, and atrial fibrosis, were decreased by T4 administration.^182^ The cross-sectional area and diameter of LA myocytes were reduced in hypothyroid and increased in hyperthyroid rats.^182^ While some studies reported an association of hypothyroidism with LA remodelling (marked by increased LA diameter) and increased preoperative AF incidence in patients with heart valvular disease^183^ or dilated cardiomyopathy,^184^ others did not confirm this.^185^^,^^186^ The impaired ejection fraction, presence of multiple valvular lesions, and a lower recovery rate of LA enlargement after valve surgery were also observed in hypothyroidism.^183^ Overt and subclinical hypothyroidism also increased the risk of post-operative-AF in the patients after cardiac surgery^187–189^ At the molecular level, hypothyroidism was associated with increased serum levels of CRP, TNF-1α, IL-6, and TGF-β1 in rats, induced secretion of the cardiac stress markers ANP, brain natriuretic peptide (BNP) (regulated by TH) and cardiac troponin-T.^175^ Hyperthyroidism also caused increase in cardiac TGF-β1 in cardiac hypertrophy mediated by angiotensin-II receptors^190^ and was associated with increased protein and ribosome synthesis.^191^^,^^192^ These observations suggest that TH are important regulators of cardiac remodelling (*Figure 6*).

## 8. Calcitonin and AF

Calcitonin (CT) is canonically secreted by parafollicular cells (C cells) of the thyroid gland and is a 32 amino acid single-chain peptide that is cleaved from a precursor pro-CT by protein convertases.^193^ Human CT originates from the CT-related polypeptide-alpha (*CALCA*) gene on chromosome-11 (ID: ENSG00000110680) that also encodes alpha-calcitonin gene-related peptide (αCGRP, a potent vasodilator with functions in the nervous and vascular systems).^194^

CT plays a well-known role in bone metabolism^195^ and plasma Ca^2+^-homeostasis.^196^ Extra-thyroidal CT expression is present in organs, such as the brain, uterus, prostate, and central nervous system.^197^^,^^198^ We recently discovered that atrial cardiomyocytes actively produce CT.^199^ Regardless of where CT is produced, it exerts its effects via binding to the CT receptor (CTR),^200^ the seven-transmembrane domain class II (family B) G protein-coupled receptor that can couple to Gs, Gi, or Gq proteins.^201^^,^^202^ The CTRs are expressed in tissues such as kidneys,^203^, osteoclasts,^204^ skeletal muscle,^205^ and recently identified in human atrial fibroblasts.^199^ A wide distribution of the CT and CTR indicates that CT-CTR signalling may be involved in the (patho)physiology of multiple systems, including the heart.

The role of the CT-CTR cascade in AF is unclear, however, key risk factors for AF, age^206^ and BMI,^207^ are associated with decreased circulating CT-levels and CTR single-nucleotide polymorphisms respectively.^208^^,^^209^ Early work in dog and rabbit models of Ca^2+^-induced arrhythmias observed antiarrhythmic effects of CT on Ca^2+^-induced ventricular arrhythmias^210^ and the inhibition of atrial chrono-/inotropic function.^211^ The mechanisms of these effects are unknown, though studies in non-cardiac cells showed that CT affected ion fluxes (e.g. neuronal Ca^2+^-currents,^212^ kidney Ca^2+^-channels and NCX^213^ and, intracellular Ca^2+214,215^ and implicated in AF pathogenesis^216^ mitochondrial Ca^2+^ influx,^217^ in CT-secreting cells) and channel expression (e.g. neuronal Na_V_1.3, Na_V_1.8, and Na_V_1.9^218^). RA cardiomyocytes from patients with persistent AF secrete six-fold less CT compared to sinus-rhythm controls.^199^ Knockdown (KD) of atrial cardiomyocyte-CT in an atrial-specific LKB1-KD model of spontaneous AF caused ∼three-fold higher incidence and ∼16-fold longer duration of spontaneous AF episodes, which commenced at a younger age vs. control LKB1-KD mice.^199^ Overexpression of CT in atrial cardiomyocytes of the LKB1-KD mice prevented atrial arrhythmia.^199^ Global deletion of the CTR in mice resulted in increased AF-inducibility.^199^ These findings point to a potentially important relationship between the cardiac CT-CTR axis and AF arrhythmogenesis.

**Figure 7:**
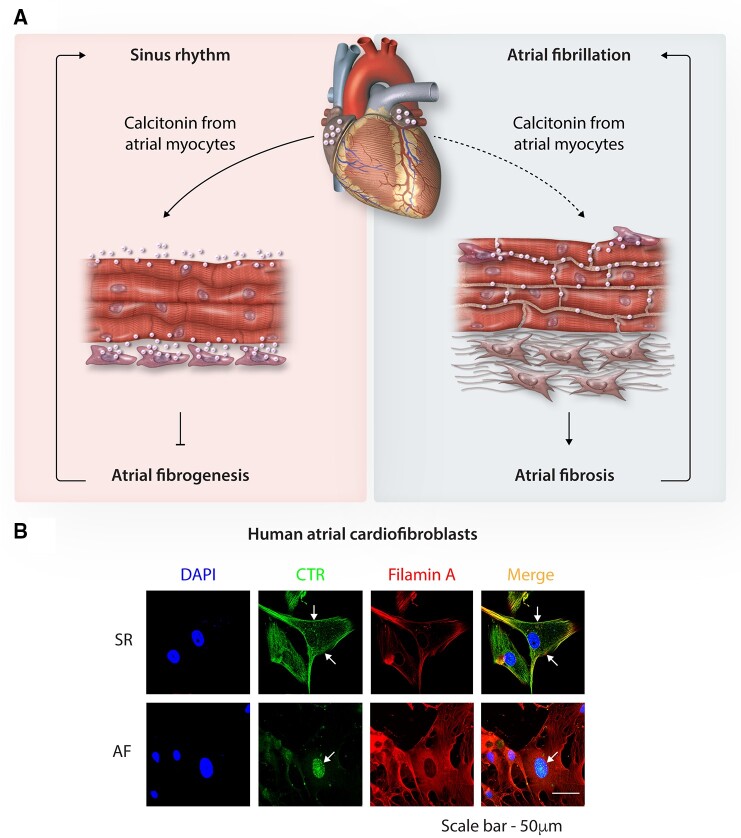
Calcitonin signalling and AF-induced remodelling. (*A*) In healthy hearts, atrial cardiomyocytes secrete calcitonin, which binds to the calcitonin-receptors (CTRs) on atrial fibroblasts, controlling extracellular matrix deposition and helping to maintain normal sinus rhythm. In AF, calcitonin signalling is disordered by reduced secretion of calcitonin by atrial cardiomyocytes and reduced calcitonin-receptor responsiveness; these changes impede the calcitonin-mediated brake on fibrogenesis causing atrial fibrosis and increased arrhythmogenesis (created with *Biorender.com*). (*B*) CTRs (green) in atrial fibroblasts co-stained with filamin A (red) and 4′,6-diamidino-2-phenylindole (DAPI) (blue). Relocalization of CTRs from the cell membrane to the nucleus explains CTR hyporesponsiveness. Scale bar = 50 μm; adapted from Moreira *et al.*^199^

CT-CTR signalling is important for tissue fibrogenesis, as it regulates collagen homeostasis, e.g., CT inhibits collagen breakdown in bones^197^ and in chondrocytes,^219^ while CTR-cKO promotes fibrosis in murine skeletal muscle.^220^ Binding of cardiac CT to the surface CTRs of neighbouring human atrial fibroblasts inhibits the production of ECM proteins like collagens-I/III, TIMP2, and SERPIN.^199^ In patients with persistent AF, accompanied by significant fibrotic remodelling, fibroblast-CTR localization was altered and confined to the intracellular space, thus, precluding CT from binding CTR (*Figure 7A*). CTR-KO mice or atrial-specific cardiomyocyte-CT-KD in the LKB1-KD mice exacerbated atrial fibrosis, suppressed by overexpression of CT in atrial cardiomycytes.^199^ In the light of this work (*Figure 7B*), maintaining CT-CTR signalling in atrial myocardium may benefit patients with AF.

## 9. Towards ‘hormonal therapeutics’ in AF

The evidence discussed in this review unequivocally demonstrates a potent regulatory role of both endocrine and cardiac para-/autocrine systems on myocardial function and structure in AF. Although currently not performed, screening of patients with AF for both systemic and cardiac (when possible) hormonal imbalance (in combination with other conventionally used diagnostic tests) may potentially advance treatment-stratification and existing treatment options with the new hormone-based therapies.

Such therapies would first of all aim to treat the prime underlying endocrine pathology (e.g. diabetes, pheochromocytoma, and obesity) with conventional therapies to reduce the risk of new-onset AF or prevent arrhythmia progression. In some cases, available therapies can fully cure the underlying endocrine disorder (e.g. resection of pheochromocytoma) and, hence, reduce risk of AF. Current treatment options for common endocrine conditions (e.g. diabetes and obesity) are often suboptimal; nevertheless, they help to reduce, while not completely eliminating, the risk of AF onset and progression. Furthermore, contemporary medications with improved safety profile may offer improved possibilities for control of AF, for example, with sodium-glucose co-transporter-2 inhibitors that may reduce risk of AF in T2DM,^221^ or with glucagon-like peptide-1 receptor agonists (except albiglutide^222^) that advantageously do not increase risk of AF in obese patients with DM.^223^ Looking into the future, control of metabolism with, for example, modified synthetic secreted factors or inhibitors may not only improve cardiometabolic conditions like DM and hypertension^224^ but may also hold promise to control AF risks associated with these serious pathologies.

Some endocrine therapies targeting selective pathways, e.g., inflammation and fibrosis, are likely to aid treatment of specific type(s) of AF, e.g., corticosteroids, due to their potent anti-inflammatory properties, might reduce AF recurrence after ablation procedures^225^ and incidence of post-operative AF.^226^ Selective inhibition of inflammation, for instance with GILZ small molecules, may help to circumvent undesirable side-effects of broad-spectrum glucocorticoids and improve therapeutic options in AF.^13^

Availability of the RAAS antagonists [angiotensin-II-converting enzymes inhibitors and angiotensin II receptor blockers (ARB)], owing to their pronounced antifibrotic effects, may not only control blood pressure in hypertensive patients but to also reduce the occurrence, development, and duration of AF.^227^ A recent meta-analysis of 7914 patients showed that aldosterone pathway blockade with MR antagonists limited AF recurrence and, to a lesser extent, prevented the new onset of AF.^228^ Since NPs counter-balance RAAS, recombinant human NPs (e.g. nesiritide—recombinant BNP) combined with neprilysin inhibitors (e.g. sacubitril, enhancing NP signalling) and ARBs (e.g. valsartan), denoted ARNi (currently approved for the heart failure management)^228^ or synthetic modified NPs designed to preferentially enhance signalling via specific NPRs, may also have potential benefits for AF management.

Sex hormone (testosterone and oestrogen) replacement therapies in patients with AF may also help to prevent AF.^229^^,^^230^ However, altering sex hormone-dependent pathways may increase the risk of stroke, cardiac arrest, and life-threatening ventricular arrhythmias (due to altered ventricular repolarization and prolonged QT interval).^231^^,^^232^ Thus, potential risks of such therapies should be carefully balanced against their benefits.

TH replacement therapy can also be beneficial.^233^^,^^234^ Disrupted CT-CTR signalling in AF might be amenable to CT-based therapies used to treat conditions like osteoporosis and Paget’s disease. However, their use is limited due to a release of anti-CT antibodies in some patients and CTR internalization during prolonged CT treatment.^235^ Gene-therapy to overexpress CT in atrial cardiomyocytes might offer a tool to manipulate CT levels in a controlled manner. Patients with persistent AF do not maintain membrane CTR localization (*Figure 7A*)^199^; thus, strategies to normalize localization of the CTR is a necessary and challenging objective in attempts to exploit CT-CTR signalling to prevent atrial structural remodelling in AF-patients. In addition, off-target effects of CTR-activation need to be avoided.

## 10. Conclusions

It is clear that endocrine/paracrine/autocrine effects play an important role in AF pathogenesis and might present interesting, presently underdeveloped, therapeutic targets. AF management is still very challenging, with many obstacles to optimal management.^236^ Recent discoveries, like those of cardiac CT-production and involvement in AF, novel molecular mediators (like GILZ) and the potential mechanistic role of inflammatory signalling, highlights how little we know about endocrine control of AF and how much more there is to learn in order to harness the full therapeutic potential of this critical system.

**Conflict of interest**: none declared.

### Funding

British Heart Foundation Intermediate Research Fellowship (S.R.); Canadian Institute of Health Research (CIHR) post-doc fellowship (A.T.); the Canadian Institutes of Health Research (CIHR) PJT 166105 and MOP142486 and The Heart and Stroke Foundation of Canada (HSFC) G-18-0022148 (R.R.); the CIHR 148401 and HSFC 18-0022032 (S.N.).

### Data availability

No new data were generated or analysed in support of this research.

## Notes

This article is part of the Spotlight Issue on Atrial Fibrillation.

## References

[1] Carpenter PC. Diagnostic evaluation of Cushing's syndrome. Endocrinol Metab Clin North Am 1988;17:445–472. 3065076

[2] van der Hooft CS , Heeringa J, Brusselle GG, Hofman A, Witteman JC, Kingma JH, Sturkenboom MC, Stricker BH. Corticosteroids and the risk of atrial fibrillation. Arch Intern Med 2006;166:1016–1020. 16682576 10.1001/archinte.166.9.1016

[3] Di Dalmazi G , Vicennati V, Pizzi C, Mosconi C, Tucci L, Balacchi C, Cosentino ER, Paolisso P, Fanelli F, Gambineri A, Pelusi C, Repaci A, Garelli S, Galie N, Borghi C, Golfieri R, Pagotto U. Prevalence and incidence of atrial fibrillation in a large cohort of adrenal incidentalomas: a long-term study. J Clin Endocrinol Metab 2020;105. 10.1210/clinem/dgaa270 32413902

[4] Neary NM , Booker OJ, Abel BS, Matta JR, Muldoon N, Sinaii N, Pettigrew RI, Nieman LK, Gharib AM. Hypercortisolism is associated with increased coronary arterial atherosclerosis: analysis of noninvasive coronary angiography using multidetector computerized tomography. J Clin Endocrinol Metab 2013;98:2045–2052. 23559084 10.1210/jc.2012-3754 PMC3644598

[5] Mancini T , Kola B, Mantero F, Boscaro M, Arnaldi G. High cardiovascular risk in patients with Cushing's syndrome according to 1999 WHO/ISH guidelines. Clin Endocrinol 2004;61:768–777. 10.1111/j.1365-2265.2004.02168.x 15579193

[6] Severinova E , Alikunju S, Deng W, Dhawan P, Sayed N, Sayed D. Glucocorticoid receptor-binding and transcriptome signature in cardiomyocytes. J Am Heart Assoc 2019;8:e011484. 30866692 10.1161/JAHA.118.011484 PMC6475044

[7] Park MH , Park SI, Kim JH, Yu J, Lee EH, Seo SR, Jo SH. The acute effects of hydrocortisone on cardiac electrocardiography, action potentials, intracellular calcium, and contraction: the role of protein kinase C. Mol Cell Endocrinol 2019;494:110488. 31207272 10.1016/j.mce.2019.110488

[8] Rao MK , Xu A, Narayanan N. Glucocorticoid modulation of protein phosphorylation and sarcoplasmic reticulum function in rat myocardium. Am J Physiol Heart Circ Physiol 2001;281:H325–33. 11406500 10.1152/ajpheart.2001.281.1.H325

[9] Lang F , Shumilina E. Regulation of ion channels by the serum- and glucocorticoid-inducible kinase SGK1. FASEB J 2013;27:3–12. 23012321 10.1096/fj.12-218230

[10] Lamothe SM , Zhang S. The serum- and glucocorticoid-inducible kinases SGK1 and SGK3 regulate hERG channel expression via ubiquitin ligase Nedd4-2 and GTPase Rab11. J Biol Chem 2013;288:15075–15084. 23589291 10.1074/jbc.M113.453670 PMC3663528

[11] Komuro J , Kaneko M, Ueda K, Nitta S, Kasao M, Shirai T. Adrenal insufficiency causes life-threatening arrhythmia with prolongation of QT interval. Heart Vessels 2016;31:1003–1005. 25771803 10.1007/s00380-015-0660-6 PMC4893060

[12] Nishizawa S , Nakamura T, Hamaoka T, Matsumuro A, Sawada T, Matsubara H. Lethal arrhythmia and corticosteroid insufficiency. Am J Emerg Med 2009;27:1167 e1–3. 10.1016/j.ajem.2008.12.001 19931775

[13] Bereshchenko O , Migliorati G, Bruscoli S, Riccardi C. Glucocorticoid-Induced leucine zipper: a novel anti-inflammatory molecule. Front Pharmacol 2019;10: 308. 30971930 10.3389/fphar.2019.00308 PMC6445858

[14] Lombardi G , Di SC, Grasso LF, Savanelli MC, Pivonello CA. R. The cardiovascular system in growth hormone excess and growth hormone deficiency. J Endocrinol Invest 2012;35:1021–1029. 23143695 10.3275/8717

[15] Higham CE , Johannsson G, Shalet SM. Hypopituitarism. Lancet 2016;388:2403–2415. 27041067 10.1016/S0140-6736(16)30053-8

[16] Rajasoorya C , Holdaway IM, Wrightson P, Scott DJ, Ibbertson HK. Determinants of clinical outcome and survival in acromegaly. Clin Endocrinol (Oxf) 1994;41:95–102. 8050136 10.1111/j.1365-2265.1994.tb03789.x

[17] Castellano G , Affuso F, Conza PD, Fazio S. The GH/IGF-1 axis and heart failure. Curr Cardiol Rev 2009;5:203–215. 20676279 10.2174/157340309788970306 PMC2822143

[18] Lu C , Schwartzbauer G, Sperling MA, Devaskar SU, Thamotharan S, Robbins PD, McTiernan CF, Liu JL, Jiang J, Frank SJ, Menon RK. Demonstration of direct effects of growth hormone on neonatal cardiomyocytes. J Biol Chem 2001;276:22892–22900. 11303022 10.1074/jbc.M011647200

[19] Tadros R , Ton AT, Fiset C, Nattel S. Sex differences in cardiac electrophysiology and clinical arrhythmias: epidemiology, therapeutics, and mechanisms. Can J Cardiol 2014;30:783–792. 24970790 10.1016/j.cjca.2014.03.032

[20] Gagliano-Jucá T , Basaria S. Testosterone replacement therapy and cardiovascular risk. Nat Rev Cardiol 2019;16:555–574. 31123340 10.1038/s41569-019-0211-4

[21] Magnani JW , Moser CB, Murabito JM, Sullivan LM, Wang N, Ellinor PT, Vasan RS, Benjamin EJ, Coviello AD. Association of sex hormones, aging, and atrial fibrillation in men: the Framingham Heart Study. Circ Arrhythm Electrophysiol 2014;7:307–312. 24610804 10.1161/CIRCEP.113.001322 PMC4035016

[22] Wamboldt R , Haseeb S, Waddington A, Baranchuk A. Cardiac arrhythmias secondary to hormone therapy in trans women. Expert Rev Cardiovasc Ther 2019;17:335–343. 30987471 10.1080/14779072.2019.1606713

[23] Pastuszak AW , Kohn TP, Estis J, Lipshultz LI. Low plasma testosterone is associated with elevated cardiovascular disease biomarkers. J Sex Med 2017;14:1095–1103. 28757119 10.1016/j.jsxm.2017.06.015 PMC5718197

[24] Tsuneda T , Yamashita T, Kato T, Sekiguchi A, Sagara K, Sawada H, Aizawa T, Fu LT, Fujiki A, Inoue H. Deficiency of testosterone associates with the substrate of atrial fibrillation in the rat model. J Cardiovasc Electrophysiol 2009;20:1055–1060. 19460074 10.1111/j.1540-8167.2009.01474.x

[25] Zhang Y , Wang HM, Wang YZ, Zhang YY, Jin XX, Zhao Y, Wang J, Sun YL, Xue GL, Li PH, Huang QH, Yang BF, Pan ZW. Increment of late sodium currents in the left atrial myocytes and its potential contribution to increased susceptibility of atrial fibrillation in castrated male mice. Heart Rhythm 2017;14:1073–1080. 28185917 10.1016/j.hrthm.2017.01.046

[26] Tsai WC , Yang LY, Chen YC, Kao YH, Lin YK, Chen SA, Cheng CF, Chen YJ. Ablation of the androgen receptor gene modulates atrial electrophysiology and arrhythmogenesis with calcium protein dysregulation. Endocrinology 2013;154:2833–2842. 23748361 10.1210/en.2012-2265

[27] Briet M , Schiffrin EL. Aldosterone: effects on the kidney and cardiovascular system. Nat Rev Nephrol 2010;6:261–273. 20234356 10.1038/nrneph.2010.30

[28] Rossi GP , Bernini G, Caliumi C, Desideri G, Fabris B, Ferri C, Ganzaroli C, Giacchetti G, Letizia C, Maccario M, Mallamaci F, Mannelli M, Mattarello MJ, Moretti A, Palumbo G, Parenti G, Porteri E, Semplicini A, Rizzoni D, Rossi E, Boscaro M, Pessina AC, Mantero F, PAPY Study Investigators. A prospective study of the prevalence of primary aldosteronism in 1,125 hypertensive patients. J Am Coll Cardiol 2006;48:2293–2300. 17161262 10.1016/j.jacc.2006.07.059

[29] Milliez P , Girerd X, Plouin PF, Blacher J, Safar ME, Mourad JJ. Evidence for an increased rate of cardiovascular events in patients with primary aldosteronism. J Am Coll Cardiol 2005;45:1243–1248. 15837256 10.1016/j.jacc.2005.01.015

[30] Rossi GP , Maiolino G, Flego A, Belfiore A, Bernini G, Fabris B, Ferri C, Giacchetti G, Letizia C, Maccario M, Mallamaci F, Muiesan ML, Mannelli M, Negro A, Palumbo G, Parenti G, Rossi E, Mantero F. PAPY Study Investigators. Adrenalectomy lowers incident atrial fibrillation in primary aldosteronism patients at long term. Hypertension 2018;71:585–591. 29483224 10.1161/HYPERTENSIONAHA.117.10596

[31] Swedberg K , Zannad F, McMurray JJ, Krum H, van Veldhuisen DJ, Shi H, Vincent J, Pitt B, EMPHASIS-HF Study Investigators. Eplerenone and atrial fibrillation in mild systolic heart failure: results from the EMPHASIS-HF (Eplerenone in Mild Patients Hospitalization And SurvIval Study in Heart Failure) study. J Am Coll Cardiol 2012;59:1598–1603. 22538330 10.1016/j.jacc.2011.11.063

[32] Sun Y , Ramires FJ, Weber KT. Fibrosis of atria and great vessels in response to angiotensin II or aldosterone infusion. Cardiovasc Res 1997;35:138–147. 9302358 10.1016/s0008-6363(97)00097-7

[33] Fiebeler A , Schmidt F, Muller DN, Park JK, Dechend R, Bieringer M, Shagdarsuren E, Breu V, Haller H, Luft FC. Mineralocorticoid receptor affects AP-1 and nuclear factor-kappab activation in angiotensin II-induced cardiac injury. Hypertension 2001;37:787–793. 11230374 10.1161/01.hyp.37.2.787

[34] Yoshida K , Kim-Mitsuyama S, Wake R, Izumiya Y, Izumi Y, Yukimura T, Ueda M, Yoshiyama M, Iwao H. Excess aldosterone under normal salt diet induces cardiac hypertrophy and infiltration via oxidative stress. Hypertens Res 2005;28:447–455. 16156509 10.1291/hypres.28.447

[35] Oestreicher EM , Martinez-Vasquez D, Stone JR, Jonasson L, Roubsanthisuk W, Mukasa K, Adler GK. Aldosterone and not plasminogen activator inhibitor-1 is a critical mediator of early angiotensin II/NG-nitro-L-arginine methyl ester-induced myocardial injury. Circulation 2003;108:2517–2523. 14581407 10.1161/01.CIR.0000097000.51723.6F

[36] Shen JZ , Morgan J, Tesch GH, Rickard AJ, Chrissobolis S, Drummond GR, Fuller PJ, Young MJ. Cardiac tissue injury and remodeling is dependent upon MR regulation of activation pathways in cardiac tissue macrophages. Endocrinology 2016;157:3213–3223. 27253999 10.1210/en.2016-1040

[37] Keidar S , Kaplan M, Pavlotzky E, Coleman R, Hayek T, Hamoud S, Aviram M. Aldosterone administration to mice stimulates macrophage NADPH oxidase and increases atherosclerosis development: a possible role for angiotensin-converting enzyme and the receptors for angiotensin II and aldosterone. Circulation 2004;109:2213–2220. 15123520 10.1161/01.CIR.0000127949.05756.9D

[38] Yang Y , Chen S, Tao L, Gan S, Luo H, Xu Y, Shen X. Inhibitory effects of oxymatrine on transdifferentiation of neonatal rat cardiac fibroblasts to myofibroblasts induced by aldosterone via keap1/nrf2 signaling pathways in vitro. Med Sci Monit 2019;25:5375–5388. 31325292 10.12659/MSM.915542 PMC6662943

[39] Reil JC , Hohl M, Selejan S, Lipp P, Drautz F, Kazakow A, Munz BM, Muller P, Steendijk P, Reil GH, Allessie MA, Bohm M, Neuberger HR. Aldosterone promotes atrial fibrillation. Eur Heart J 2012;33:2098–2108. 21816854 10.1093/eurheartj/ehr266

[40] Lammers C , Dartsch T, Brandt MC, Rottlander D, Halbach M, Peinkofer G, Ockenpoehler S, Weiergraeber M, Schneider T, Reuter H, Muller-Ehmsen J, Hescheler J, Hoppe UC, Zobel C. Spironolactone prevents aldosterone induced increased duration of atrial fibrillation in rat. Cell Physiol Biochem 2012;29:833–840. 22613983 10.1159/000178483

[41] Ouvrard-Pascaud A , Sainte-Marie Y, Benitah JP, Perrier R, Soukaseum C, Nguyen Dinh Cat A, Royer A, Le Quang K, Charpentier F, Demolombe S, Mechta-Grigoriou F, Beggah AT, Maison-Blanche P, Oblin ME, Delcayre C, Fishman GI, Farman N, Escoubet B, Jaisser F. Conditional mineralocorticoid receptor expression in the heart leads to life-threatening arrhythmias. Circulation 2005;111:3025–3033. 15939817 10.1161/CIRCULATIONAHA.104.503706 PMC3635833

[42] Benitah JP , Vassort G. Aldosterone upregulates Ca(2+) current in adult rat cardiomyocytes. Circ Res 1999;85:1139–1145. 10590240 10.1161/01.res.85.12.1139

[43] Perrier R , Richard S, Sainte-Marie Y, Rossier BC, Jaisser F, Hummler E, Bénitah J-P. A direct relationship between plasma aldosterone and cardiac L-type Ca2+ current in mice. J Physiol 2005;569:153–162. 16166161 10.1113/jphysiol.2005.092692 PMC1464196

[44] Gomez AM , Rueda A, Sainte-Marie Y, Pereira L, Zissimopoulos S, Zhu X, Schaub R, Perrier E, Perrier R, Latouche C, Richard S, Picot MC, Jaisser F, Lai FA, Valdivia HH, Benitah JP. Mineralocorticoid modulation of cardiac ryanodine receptor activity is associated with downregulation of FK506-binding proteins. Circulation 2009;119:2179–2187. 19364981 10.1161/CIRCULATIONAHA.108.805804

[45] Lavall D , Selzer C, Schuster P, Lenski M, Adam O, Schafers HJ, Bohm M, Laufs U. The mineralocorticoid receptor promotes fibrotic remodeling in atrial fibrillation. J Biol Chem 2014;289:6656–6668. 24469458 10.1074/jbc.M113.519256 PMC3945327

[46] Tsai CT , Chiang FT, Tseng CD, Hwang JJ, Kuo KT, Wu CK, Yu CC, Wang YC, Lai LP, Lin JL. Increased expression of mineralocorticoid receptor in human atrial fibrillation and a cellular model of atrial fibrillation. J Am Coll Cardiol 2010;55:758–770. 20170814 10.1016/j.jacc.2009.09.045

[47] Prejbisz A , Lenders JW, Eisenhofer G, Januszewicz A. Cardiovascular manifestations of phaeochromocytoma. J Hypertens 2011;29:2049–2060. 21826022 10.1097/HJH.0b013e32834a4ce9

[48] Nazari MA , Rosenblum JS, Haigney MC, Rosing DR, Pacak K. Pathophysiology and acute management of tachyarrhythmias in pheochromocytoma: JACC review topic of the week. J Am Coll Cardiol 2020;76:451–464. 32703516 10.1016/j.jacc.2020.04.080 PMC7454044

[49] Scheja L , Heeren J. The endocrine function of adipose tissues in health and cardiometabolic disease. Nat Rev Endocrinol 2019;15:507–524. 31296970 10.1038/s41574-019-0230-6

[50] Pathak RK , Mahajan R, Lau DH, Sanders P. The implications of obesity for cardiac arrhythmia mechanisms and management. Can J Cardiol 2015;31:203–210. 25661555 10.1016/j.cjca.2014.10.027

[51] Nattel S. Atrial fibrillation and body composition: is it fat or lean that ultimately determines the risk? J Am Coll Cardiol 2017;69:2498–2501. 28521887 10.1016/j.jacc.2017.03.566

[52] Fenger-Grøn M , Overvad K, Tjønneland A, Frost L. Lean body mass is the predominant anthropometric risk factor for atrial fibrillation. J Am Coll Cardiol 2017;69:2488–2497. 28521886 10.1016/j.jacc.2017.03.558

[53] Shuai W , Kong B, Fu H, Shen C, Jiang X, Huang H. MD1 deficiency promotes inflammatory atrial remodelling induced by high-fat diets. Can J Cardiol 2019;35:208–216. 30760428 10.1016/j.cjca.2018.11.020

[54] Yao C , Veleva T, Scott L Jr, Cao S, Li L, Chen G, Jeyabal P, Pan X, Alsina KM, Abu-Taha ID, Ghezelbash S, Reynolds CL, Shen YH, LeMaire SA, Schmitz W, Muller FU, El-Armouche A, Tony Eissa N, Beeton C, Nattel S, Wehrens XHT, Dobrev D, Li N. Enhanced cardiomyocyte NLRP3 inflammasome signaling promotes atrial fibrillation. Circulation 2018;138:2227–2242. 29802206 10.1161/CIRCULATIONAHA.118.035202 PMC6252285

[55] Ghanim H , Aljada A, Hofmeyer D, Syed T, Mohanty P, Dandona P. Circulating mononuclear cells in the obese are in a proinflammatory state. Circulation 2004;110:1564–1571. 15364812 10.1161/01.CIR.0000142055.53122.FA

[56] Musaad S , Haynes EN. Biomarkers of obesity and subsequent cardiovascular events. Epidemiol Rev 2007;29:98–114. 17494057 10.1093/epirev/mxm005 PMC4682894

[57] Abed HS , Samuel CS, Lau DH, Kelly DJ, Royce SG, Alasady M, Mahajan R, Kuklik P, Zhang Y, Brooks AG, Nelson AJ, Worthley SG, Abhayaratna WP, Kalman JM, Wittert GA, Sanders P. Obesity results in progressive atrial structural and electrical remodeling: implications for atrial fibrillation. Heart Rhythm 2013;10:90–100. 23063864 10.1016/j.hrthm.2012.08.043

[58] Nalliah CJ , Bell JR, Raaijmakers AJA, Waddell HM, Wells SP, Bernasochi GB, Montgomery MK, Binny S, Watts T, Joshi SB, Lui E, Sim CB, Larobina M, O'Keefe M, Goldblatt J, Royse A, Lee G, Porrello ER, Watt MJ, Kistler PM, Sanders P, Delbridge LMD, Kalman JM. Epicardial adipose tissue accumulation confers atrial conduction abnormality. J Am Coll Cardiol 2020;76:1197–1211. 32883413 10.1016/j.jacc.2020.07.017

[59] Hatem SN , Redheuil A, Gandjbakhch E. Cardiac adipose tissue and atrial fibrillation: the perils of adiposity. Cardiovasc Res 2016;109:502–509. 26790475 10.1093/cvr/cvw001

[60] Fukui A , Takahashi N, Nakada C, Masaki T, Kume O, Shinohara T, Teshima Y, Hara M, Saikawa T. Role of leptin signaling in the pathogenesis of angiotensin II-mediated atrial fibrosis and fibrillation. Circ Arrhythm Electrophysiol 2013;6:402–409. 23406575 10.1161/CIRCEP.111.000104

[61] Goodfriend TL , Kelley DE, Goodpaster BH, Winters SJ. Visceral obesity and insulin resistance are associated with plasma aldosterone levels in women. Obes Res 1999;7:355–362. 10440591 10.1002/j.1550-8528.1999.tb00418.x

[62] Ermakov S , Azarbal F, Stefanick ML, LaMonte MJ, Li W, Tharp KM, Martin LW, Nassir R, Salmoirago-Blotcher E, Albert CM, Manson JE, Assimes TL, Hlatky MA, Larson JC, Perez MV. The associations of leptin, adiponectin and resistin with incident atrial fibrillation in women. Heart 2016;102:1354–1362. 27146694 10.1136/heartjnl-2015-308927

[63] Packer M. Leptin-aldosterone-neprilysin axis: identification of its distinctive role in the pathogenesis of the three phenotypes of heart failure in people with obesity. Circulation 2018;137:1614–1631. 29632154 10.1161/CIRCULATIONAHA.117.032474

[64] Standeven KF , Hess K, Carter AM, Rice GI, Cordell PA, Balmforth AJ, Lu B, Scott DJ, Turner AJ, Hooper NM, Grant PJ. Neprilysin, obesity and the metabolic syndrome. Int J Obes 2011;35:1031–1040. 10.1038/ijo.2010.227 PMC3040694 21042321

[65] Schling P , Schafer T. Human adipose tissue cells keep tight control on the angiotensin II levels in their vicinity. J Biol Chem 2002;277:48066–48075. 12196514 10.1074/jbc.M204058200

[66] Bentley-Lewis R , Adler GK, Perlstein T, Seely EW, Hopkins PN, Williams GH, Garg R. Body mass index predicts aldosterone production in normotensive adults on a high-salt diet. J Clin Endocrinol Metab 2007;92:4472–4475. 17726083 10.1210/jc.2007-1088 PMC4428584

[67] Munger TM , Dong YX, Masaki M, Oh JK, Mankad SV, Borlaug BA, Asirvatham SJ, Shen WK, Lee HC, Bielinski SJ, Hodge DO, Herges RM, Buescher TL, Wu JH, Ma C, Zhang Y, Chen PS, Packer DL, Cha YM. Electrophysiological and hemodynamic characteristics associated with obesity in patients with atrial fibrillation. J Am Coll Cardiol 2012;60:851–860. 22726633 10.1016/j.jacc.2012.03.042

[68] Gumprecht J , Domek M, Lip GYH, Shantsila A. Invited review: hypertension and atrial fibrillation: epidemiology, pathophysiology, and implications for management. J Hum Hypertens 2019;33:824–836. 31690818 10.1038/s41371-019-0279-7

[69] Jansen HJ , Bohne LJ, Gillis AM, Rose RA. Atrial remodeling and atrial fibrillation in acquired forms of cardiovascular disease. Heart Rhythm 2020;1:147–159. 10.1016/j.hroo.2020.05.002 PMC8183954 34113869

[70] Heijman J , Voigt N, Nattel S, Dobrev D. Cellular and molecular electrophysiology of atrial fibrillation initiation, maintenance, and progression. Circ Res 2014;114:1483–1499. 24763466 10.1161/CIRCRESAHA.114.302226

[71] Kornej J , Borschel CS, Benjamin EJ, Schnabel RB. Epidemiology of atrial fibrillation in the 21st century: novel methods and new insights. Circ Res 2020;127:4–20. 32716709 10.1161/CIRCRESAHA.120.316340 PMC7577553

[72] Forrester SJ , Booz GW, Sigmund CD, Coffman TM, Kawai T, Rizzo V, Scalia R, Eguchi S. Angiotensin II signal transduction: an update on mechanisms of physiology and pathophysiology. Physiol Rev 2018;98:1627–1738. 29873596 10.1152/physrev.00038.2017 PMC6335102

[73] Seccia TM , Caroccia B, Muiesan ML, Rossi GP. Atrial fibrillation and arterial hypertension: a common duet with dangerous consequences where the renin angiotensin-aldosterone system plays an important role. Int J Cardiol 2016;206:71–76. 26774837 10.1016/j.ijcard.2016.01.007

[74] Ciaroni S , Cuenoud L, Bloch A. Clinical study to investigate the predictive parameters for the onset of atrial fibrillation in patients with essential hypertension. Am Heart J 2000;139:814–819. 10783214 10.1016/s0002-8703(00)90012-7

[75] Jansen HJ , Mackasey M, Moghtadaei M, Belke DD, Egom EE, Tuomi JM, Rafferty SA, Kirkby AW, Rose RA. Distinct patterns of atrial electrical and structural remodeling in angiotensin II mediated atrial fibrillation. J Mol Cell Cardiol 2018;124:12–25. 30273558 10.1016/j.yjmcc.2018.09.011

[76] Mackasey M , Egom EE, Jansen HJ, Hua R, Moghtadaei M, Liu Y, Kaur J, McRae MD, Bogachev O, Rafferty SA, Ray G, Kirkby AW, Rose RA. Natriuretic peptide receptor-C protects against angiotensin II-mediated sinoatrial node disease in mice. JACC Basic Transl Sci 2018;3:824–843. 30623142 10.1016/j.jacbts.2018.08.004 PMC6314975

[77] Jansen HJ , Mackasey M, Moghtadaei M, Liu Y, Kaur J, Egom EE, Tuomi JM, Rafferty SA, Kirkby AW, Rose RA. NPR-C (natriuretic peptide receptor-C) modulates the progression of angiotensin II-mediated atrial fibrillation and atrial remodeling in mice. Circ Arrhythm Electrophysiol 2019;12:e006863. 30636477 10.1161/CIRCEP.118.006863

[78] Purohit A , Rokita AG, Guan X, Chen B, Koval OM, Voigt N, Neef S, Sowa T, Gao Z, Luczak ED, Stefansdottir H, Behunin AC, Li N, El-Accaoui RN, Yang B, Swaminathan PD, Weiss RM, Wehrens XH, Song LS, Dobrev D, Maier LS, Anderson ME. Oxidized Ca2+/calmodulin-dependent protein kinase II triggers atrial fibrillation. Circulation 2013;128:1748–1757. 24030498 10.1161/CIRCULATIONAHA.113.003313 PMC3876034

[79] Li J , Wang S, Bai J, Yang XL, Zhang YL, Che YL, Li HH, Yang YZ. Novel role for the immunoproteasome subunit PSMB10 in angiotensin ii-induced atrial fibrillation in mice. Hypertension 2018;71:866–876. 29507100 10.1161/HYPERTENSIONAHA.117.10390

[80] Choisy SC , Arberry LA, Hancox JC, James AF. Increased susceptibility to atrial tachyarrhythmia in spontaneously hypertensive rat hearts. Hypertension 2007;49:498–505. 17242301 10.1161/01.HYP.0000257123.95372.ab

[81] Pluteanu F , Heß J, Plackic J, Nikonova Y, Preisenberger J, Bukowska A, Schotten U, Rinne A, Kienitz M-C, Schäfer MK-H, Weihe E, Goette A, Kockskämper J. Early subcellular Ca2+ remodelling and increased propensity for Ca2+ alternans in left atrial myocytes from hypertensive rats. Cardiovasc Res 2015;106:87–97. 25691541 10.1093/cvr/cvv045

[82] Goette A , Bukowska A, Dobrev D, Pfeiffenberger J, Morawietz H, Strugala D, Wiswedel I, Rohl FW, Wolke C, Bergmann S, Bramlage P, Ravens U, Lendeckel U. Acute atrial tachyarrhythmia induces angiotensin II type 1 receptor-mediated oxidative stress and microvascular flow abnormalities in the ventricles. Eur Heart J 2009;30:1411–1420. 19269986 10.1093/eurheartj/ehp046 PMC2688683

[83] Swaminathan PD , Purohit A, Hund TJ, Anderson ME. Calmodulin-dependent protein kinase II: linking heart failure and arrhythmias. Circ Res 2012;110:1661–1677. 22679140 10.1161/CIRCRESAHA.111.243956 PMC3789535

[84] Moghtadaei M , Polina I, Rose RA. Electrophysiological effects of natriuretic peptides in the heart are mediated by multiple receptor subtypes. Prog Biophys Mol Biol 2016;120:37–49. 26701223 10.1016/j.pbiomolbio.2015.12.001

[85] Perrin MJ , Gollob MH. The role of atrial natriuretic peptide in modulating cardiac electrophysiology. Heart Rhythm 2012;9:610–615. 22083030 10.1016/j.hrthm.2011.11.019

[86] Springer J , Azer J, Hua R, Robbins C, Adamczyk A, McBoyle S, Bissell MB, Rose RA. The natriuretic peptides BNP and CNP increase heart rate and electrical conduction by stimulating ionic currents in the sinoatrial node and atrial myocardium following activation of guanylyl cyclase-linked natriuretic peptide receptors. J Mol Cell Cardiol 2012;52:1122–1134. 22326431 10.1016/j.yjmcc.2012.01.018

[87] Azer J , Hua R, Krishnaswamy PS, Rose RA. Effects of natriuretic peptides on electrical conduction in the sinoatrial node and atrial myocardium of the heart. J Physiol 2014;592:1025–1045. 24344164 10.1113/jphysiol.2013.265405 PMC3948561

[88] Rose RA , Lomax AE, Giles WR. Inhibition of L-type Ca2+ current by C-type natriuretic peptide in bullfrog atrial myocytes: an NPR-C-mediated effect. Am J Physiol Heart Circ Physiol 2003;285:H2454–H2462. 12881210 10.1152/ajpheart.00388.2003

[89] Potter LR , Abbey-Hosch S, Dickey DM. Natriuretic peptides, their receptors, and cyclic guanosine monophosphate-dependent signaling functions. Endocr Rev 2006;27:47–72. 16291870 10.1210/er.2005-0014

[90] Rose RA , Giles WR. Natriuretic peptide C receptor signalling in the heart and vasculature. J Physiol 2008;586:353–366. 18006579 10.1113/jphysiol.2007.144253 PMC2375602

[91] Anand-Srivastava MB. Natriuretic peptide receptor-C signaling and regulation. Peptides 2005;26:1044–1059. 15911072 10.1016/j.peptides.2004.09.023

[92] Egom EE , Vella K, Hua R, Jansen HJ, Moghtadaei M, Polina I, Bogachev O, Hurnik R, Mackasey M, Rafferty S, Ray G, Rose RA. Impaired sinoatrial node function and increased susceptibility to atrial fibrillation in mice lacking natriuretic peptide receptor C. J Physiol 2015;593:1127–1146. 25641115 10.1113/jphysiol.2014.283135 PMC4358675

[93] Hobbs A , Foster P, Prescott C, Scotland R, Ahluwalia A. Natriuretic peptide receptor-C regulates coronary blood flow and prevents myocardial ischemia/reperfusion injury: novel cardioprotective role for endothelium-derived C-type natriuretic peptide. Circulation 2004;110:1231–1235. 15337698 10.1161/01.CIR.0000141802.29945.34

[94] Moyes AJ , Chu SM, Aubdool AA, Dukinfield MS, Margulies KB, Bedi KC, Hodivala-Dilke K, Baliga RS, Hobbs AJ. C-type natriuretic peptide co-ordinates cardiac structure and function. Eur Heart J 2020;41:1006–1020. 30903134 10.1093/eurheartj/ehz093 PMC7068173

[95] Moyes AJ , Khambata RS, Villar I, Bubb KJ, Baliga RS, Lumsden NG, Xiao F, Gane PJ, Rebstock AS, Worthington RJ, Simone MI, Mota F, Rivilla F, Vallejo S, Peiro C, Sanchez Ferrer CF, Djordjevic S, Caulfield MJ, MacAllister RJ, Selwood DL, Ahluwalia A, Hobbs AJ. Endothelial C-type natriuretic peptide maintains vascular homeostasis. J Clin Invest 2014;124:4039–4051. 25105365 10.1172/JCI74281 PMC4151218

[96] Rahmutula D , Zhang H, Wilson EE, Olgin JE. Absence of natriuretic peptide clearance receptor attenuates TGF-beta1-induced selective atrial fibrosis and atrial fibrillation. Cardiovasc Res 2019;115:357–372. 30239604 10.1093/cvr/cvy224

[97] Abraham RL , Yang T, Blair M, Roden DM, Darbar D. Augmented potassium current is a shared phenotype for two genetic defects associated with familial atrial fibrillation. J Mol Cell Cardiol 2010;48:181–190. 19646991 10.1016/j.yjmcc.2009.07.020 PMC2813326

[98] Hodgson-Zingman DM , Karst ML, Zingman LV, Heublein DM, Darbar D, Herron KJ, Ballew JD, de Andrade M, Burnett JC Jr, Olson TM. Atrial natriuretic peptide frameshift mutation in familial atrial fibrillation. N Engl J Med 2008;359:158–165. 18614783 10.1056/NEJMoa0706300 PMC2518320

[99] Dickey DM , Yoder AR, Potter LR. A familial mutation renders atrial natriuretic Peptide resistant to proteolytic degradation. J Biol Chem 2009;284:19196–19202. 19458086 10.1074/jbc.M109.010777 PMC2740543

[100] Lonardo G , Cerbai E, Casini S, Giunti G, Bonacchi M, Battaglia F, Fiorani B, Stefano PL, Sani G, Mugelli A. Atrial natriuretic peptide modulates the hyperpolarization-activated current (If) in human atrial myocytes. Cardiovasc Res 2004;63:528–536. 15276478 10.1016/j.cardiores.2004.03.004

[101] Hua R , MacLeod SL, Polina I, Moghtadaei M, Jansen HJ, Bogachev O, O'Blenes SB, Sapp JL, Legare JF, Rose RA. Effects of wild-type and mutant forms of atrial natriuretic peptide on atrial electrophysiology and arrhythmogenesis. Circ Arrhythm Electrophysiol 2015;8:1240–1254. 26227000 10.1161/CIRCEP.115.002896

[102] Polonsky KS. The past 200 years in diabetes. N Engl J Med 2012;367:1332–1340. 23034021 10.1056/NEJMra1110560

[103] Warshauer JT , Bluestone JA, Anderson MS. New frontiers in the treatment of type 1 diabetes. Cell Metab 2020;31:46–61. 31839487 10.1016/j.cmet.2019.11.017 PMC6986815

[104] Kahn SE , Cooper ME, Del Prato S. Pathophysiology and treatment of type 2 diabetes: perspectives on the past, present, and future. Lancet 2014;383:1068–1083. 24315620 10.1016/S0140-6736(13)62154-6 PMC4226760

[105] Bohne LJ , Johnson D, Rose RA, Wilton SB, Gillis AM. The association between diabetes mellitus and atrial fibrillation: clinical and mechanistic insights. Front Physiol 2019;10:135. 30863315 10.3389/fphys.2019.00135 PMC6399657

[106] Hsueh W , Abel ED, Breslow JL, Maeda N, Davis RC, Fisher EA, Dansky H, McClain DA, McIndoe R, Wassef MK, Rabadan-Diehl C, Goldberg IJ. Recipes for creating animal models of diabetic cardiovascular disease. Circ Res 2007;100:1415–1427. 17525381 10.1161/01.RES.0000266449.37396.1f

[107] Polina I , Jansen HJ, Li T, Moghtadaei M, Bohne LJ, Liu Y, Krishnaswamy P, Egom EE, Belke DD, Rafferty SA, Ezeani M, Gillis AM, Rose RA. Loss of insulin signaling may contribute to atrial fibrillation and atrial electrical remodeling in type 1 diabetes. Proc Natl Acad Sci U S A 2020;117:7990–8000. 32198206 10.1073/pnas.1914853117 PMC7148583

[108] Saito S , Teshima Y, Fukui A, Kondo H, Nishio S, Nakagawa M, Saikawa T, Takahashi N. Glucose fluctuations increase the incidence of atrial fibrillation in diabetic rats. Cardiovasc Res 2014;104:5–14. 25082849 10.1093/cvr/cvu176

[109] Lin RZ , Lu Z, Anyukhovsky EP, Jiang YP, Wang HZ, Gao J, Rosen MR, Ballou LM, Cohen IS. Regulation of heart rate and the pacemaker current by phosphoinositide 3-kinase signaling. J Gen Physiol 2019;151:1051–1058. 31217223 10.1085/jgp.201812293 PMC6683667

[110] Krishnaswamy PS , Egom EE, Moghtadaei M, Jansen HJ, Azer J, Bogachev O, Mackasey M, Robbins C, Rose RA. Altered parasympathetic nervous system regulation of the sinoatrial node in Akita diabetic mice. J Mol Cell Cardiol 2015;82:125–135. 25754673 10.1016/j.yjmcc.2015.02.024

[111] Kato T , Yamashita T, Sekiguchi A, Tsuneda T, Sagara K, Takamura M, Kaneko S, Aizawa T, Fu LT. AGEs-RAGE system mediates atrial structural remodeling in the diabetic rat. J Cardiovasc Electrophysiol 2008;19:415–420. 18298515 10.1111/j.1540-8167.2007.01037.x

[112] Kato T , Yamashita T, Sekiguchi A, Tsuneda T, Sagara K, Takamura M, Kaneko S, Aizawa T, Fu LT. Angiotensin II type 1 receptor blocker attenuates diabetes-induced atrial structural remodeling. J Cardiol 2011;58:131–136. 21802905 10.1016/j.jjcc.2011.06.003

[113] Watanabe M , Yokoshiki H, Mitsuyama H, Mizukami K, Ono T, Tsutsui H. Conduction and refractory disorders in the diabetic atrium. Am J Physiol Heart Circ Physiol 2012;303:H86–95. 22561303 10.1152/ajpheart.00010.2012

[114] Zhang X , Zhang Z, Zhao Y, Jiang N, Qiu J, Yang Y, Li J, Liang X, Wang X, Tse G, Li G, Liu T. Alogliptin, a dipeptidyl peptidase-4 inhibitor, alleviates atrial remodeling and improves mitochondrial function and biogenesis in diabetic rabbits. Jaha 2017;6:10.1161/JAHA.117.005945 PMC5524117 28507060

[115] Cho NH , Shaw JE, Karuranga S, Huang Y, da Rocha Fernandes JD, Ohlrogge AW, Malanda B. IDF diabetes atlas: global estimates of diabetes prevalence for 2017 and projections for 2045. Diabetes Res Clin Pract 2018;138:271–281. 29496507 10.1016/j.diabres.2018.02.023

[116] Bell DSH , Goncalves E. Atrial fibrillation and type 2 diabetes: prevalence, etiology, pathophysiology and effect of anti-diabetic therapies. Diabetes Obes Metab 2019;21:210–217. 30144274 10.1111/dom.13512

[117] Reuter H , Gronke S, Adam C, Ribati M, Brabender J, Zobel C, Frank KF, Wippermann J, Schwinger RH, Brixius K, Muller-Ehmsen J. Sarcoplasmic Ca2+ release is prolonged in nonfailing myocardium of diabetic patients. Mol Cell Biochem 2008;308:141–149. 17952561 10.1007/s11010-007-9622-3

[118] Lamberts RR , Lingam SJ, Wang HY, Bollen IA, Hughes G, Galvin IF, Bunton RW, Bahn A, Katare R, Baldi JC, Williams MJ, Saxena P, Coffey S, Jones PP. Impaired relaxation despite upregulated calcium-handling protein atrial myocardium from type 2 diabetic patients with preserved ejection fraction. Cardiovasc Diabetol 2014;13:72. 24708792 10.1186/1475-2840-13-72 PMC3997226

[119] Sedgwick B , Riches K, Bageghni SA, O'Regan DJ, Porter KE, Turner NA. Investigating inherent functional differences between human cardiac fibroblasts cultured from nondiabetic and Type 2 diabetic donors. Cardiovasc Pathol 2014;23:204–210. 24746387 10.1016/j.carpath.2014.03.004

[120] Lubbers ER , Price MV, Mohler PJ. Arrhythmogenic substrates for atrial fibrillation in obesity. Front Physiol 2018;9:1482. 30405438 10.3389/fphys.2018.01482 PMC6204377

[121] Sanghai SR , Sardana M, Hansra B, Lessard DM, Dahlberg ST, Aurigemma GP, Fitzgibbons TP, McManus DD. Indexed left atrial adipose tissue area is associated with severity of atrial fibrillation and atrial fibrillation recurrence among patients undergoing catheter ablation. Front Cardiovasc Med 2018;5: 76. 29971239 10.3389/fcvm.2018.00076 PMC6018072

[122] Hatem SN , Sanders P. Epicardial adipose tissue and atrial fibrillation. Cardiovasc Res 2014;102:205–213. 24648445 10.1093/cvr/cvu045

[123] Haemers P , Hamdi H, Guedj K, Suffee N, Farahmand P, Popovic N, Claus P, LePrince P, Nicoletti A, Jalife J, Wolke C, Lendeckel U, Jais P, Willems R, Hatem SN. Atrial fibrillation is associated with the fibrotic remodelling of adipose tissue in the subepicardium of human and sheep atria. Eur Heart J 2017;38:53–61. 26612579 10.1093/eurheartj/ehv625

[124] Mesubi OO , Rokita AG, Abrol N, Wu Y, Chen B, Wang Q, Granger JM, Tucker-Bartley A, Luczak ED, Murphy KR, Umapathi P, Banerjee PS, Boronina TN, Cole RN, Maier LS, Wehrens XH, Pomerantz JL, Song LS, Ahima RS, Hart GW, Zachara NE, Anderson ME. Oxidized CaMKII and O-GlcNAcylation cause increased atrial fibrillation in diabetic mice by distinct mechanisms. J Clin Investig 2021;131:e95747. 10.1172/JCI95747 PMC7810480 33151911

[125] Bohne LJ , Jansen HJ, Daniel I, Dorey TW, Moghtadaei M, Belke DD, Ezeani M, Rose RA. Electrical and structural remodeling contribute to atrial fibrillation in type 2 diabetic db/db mice. Heart Rhythm 2021;18:118–129. 32911049 10.1016/j.hrthm.2020.08.019

[126] Fu L , Rao F, Lian F, Yang H, Kuang S, Wu S, Deng C, Xue Y. Mechanism of electrical remodeling of atrial myocytes and its influence on susceptibility to atrial fibrillation in diabetic rats. Life Sci 2019;239:116903. 31639397 10.1016/j.lfs.2019.116903

[127] Linz D , Hohl M, Dhein S, Ruf S, Reil JC, Kabiri M, Wohlfart P, Verheule S, Bohm M, Sadowski T, Schotten U. Cathepsin A mediates susceptibility to atrial tachyarrhythmia and impairment of atrial emptying function in Zucker diabetic fatty rats. Cardiovasc Res 2016;110:371–380. 27032673 10.1093/cvr/cvw071

[128] Kondo H , Abe I, Gotoh K, Fukui A, Takanari H, Ishii Y, Ikebe Y, Kira S, Oniki T, Saito S, Aoki K, Tanino T, Mitarai K, Kawano K, Miyoshi M, Fujinami M, Yoshimura S, Ayabe R, Okada N, Nagano Y, Akioka H, Shinohara T, Akiyoshi K, Masaki T, Teshima Y, Yufu K, Nakagawa M, Takahashi N. Interleukin 10 treatment ameliorates high-fat diet-induced inflammatory atrial remodeling and fibrillation. Circ Arrhythm Electrophysiol 2018;11:e006040. 29748196 10.1161/CIRCEP.117.006040

[129] Chan YH , Chang GJ, Lai YJ, Chen WJ, Chang SH, Hung LM, Kuo CT, Yeh YH. Atrial fibrillation and its arrhythmogenesis associated with insulin resistance. Cardiovasc Diabetol 2019;18:125. 31558158 10.1186/s12933-019-0928-8 PMC6761716

[130] Fender AC , Kleeschulte S, Stolte S, Leineweber K, Kamler M, Bode J, Li N, Dobrev D. Thrombin receptor PAR4 drives canonical NLRP3 inflammasome signaling in the heart. Basic Res Cardiol 2020;115:10. 31912235 10.1007/s00395-019-0771-9 PMC7384378

[131] Fukui A , Ikebe-Ebata Y, Kondo H, Saito S, Aoki K, Fukunaga N, Shinohara T, Masaki T, Teshima Y, Takahashi N. Hyperleptinemia exacerbates high-fat diet-mediated atrial fibrosis and fibrillation. J Cardiovasc Electrophysiol 2017;28:702–710. 28257569 10.1111/jce.13200

[132] Jabbar A , Pingitore A, Pearce SH, Zaman A, Iervasi G, Razvi S. Thyroid hormones and cardiovascular disease. Nat Rev Cardiol 2017;14:39–55. 27811932 10.1038/nrcardio.2016.174

[133] Sawin CT , Geller A, Wolf PA, Belanger AJ, Baker E, Bacharach P, Wilson PW, Benjamin EJ, D'Agostino RB. Low serum thyrotropin concentrations as a risk factor for atrial fibrillation in older persons. N Engl J Med 1994;331:1249–1252. 7935681 10.1056/NEJM199411103311901

[134] Auer J , Eber B. [ Subclinical hyperthyroidism and atrial fibrillation. Acta Med Austriaca 2003;30:98–99. ]. 14710478

[135] Gammage MD , Parle JV, Holder RL, Roberts LM, Hobbs FD, Wilson S, Sheppard MC, Franklyn JA. Association between serum free thyroxine concentration and atrial fibrillation. Arch Intern Med 2007;167:928–934. 17502534 10.1001/archinte.167.9.928

[136] Krahn AD , Klein GJ, Kerr CR, Boone J, Sheldon R, Green M, Talajic M, Wang X, Connolly S. How useful is thyroid function testing in patients with recent-onset atrial fibrillation? The Canadian Registry of Atrial Fibrillation Investigators. Arch Internal Med 1996;156:2221–2224. 8885821

[137] Cohen-Lehman J , Dahl P, Danzi S, Klein I. Effects of amiodarone therapy on thyroid function. Nat Rev Endocrinol 2010;6:34–41. 19935743 10.1038/nrendo.2009.225

[138] von Olshausen K , Bischoff S, Kahaly G, Mohr-Kahaly S, Erbel R, Beyer J, Meyer J. Cardiac arrhythmias and heart rate in hyperthyroidism. Am J Cardiol 1989;63:930–933. 2929466 10.1016/0002-9149(89)90142-2

[139] Wustmann K , Kucera JP, Zanchi A, Burow A, Stuber T, Chappuis B, Diem P, Delacrétaz E. Activation of electrical triggers of atrial fibrillation in hyperthyroidism. The Journal of Clinical Endocrinology and Metabolism 2008;93:2104–2108. 18349059 10.1210/jc.2008-0092

[140] Venkatesh N , Lynch JJ, Uprichard AC, Kitzen JM, Singh BN, Lucchesi BR. Hypothyroidism renders protection against lethal ventricular arrhythmias in a conscious canine model of sudden death. J Cardiovasc Pharmacol 1991;18:703–710. 1723767 10.1097/00005344-199111000-00008

[141] Razvi S , Jabbar A, Pingitore A, Danzi S, Biondi B, Klein I, Peeters R, Zaman A, Iervasi G. Thyroid hormones and cardiovascular function and diseases. J Am Coll Cardiol 2018;71:1781–1796. 29673469 10.1016/j.jacc.2018.02.045

[142] Davis PJ , Goglia F, Leonard JL. Nongenomic actions of thyroid hormone. Nat Rev Endocrinol 2016;12:111–121. 26668118 10.1038/nrendo.2015.205

[143] Biondi B. Mechanisms in endocrinology: heart failure and thyroid dysfunction. Eur J Endocrinol 2012;167:609–618. 22956554 10.1530/EJE-12-0627

[144] Arnsdorf MF , Childers RW. Atrial electrophysiology in experimental hyperthyroidism in rabbits. Circ Res 1970;26:575–581. 4191904 10.1161/01.res.26.5.575

[145] Johnson PN , Freedberg AS, Marshall JM. Action of thyroid hormone on the transmembrane potentials from sinoatrial node cells and atrial muscle cells in isolated atria of rabbits. Cardiology 1973;58:273–289. 4792097 10.1159/000169643

[146] Freedberg AS , Papp JG, Williams EM. The effect of altered thyroid state on atrial intracellular potentials. J Physiol 1970;207:357–369. 5499024 10.1113/jphysiol.1970.sp009066 PMC1348711

[147] Sharp NA , Neel DS, Parsons RL. Influence of thyroid hormone levels on the electrical and mechanical properties of rabbit papillary muscle. J Mol Cell Cardiol 1985;17:119–132. 2987515 10.1016/s0022-2828(85)80015-8

[148] Binah O , Rubinstein I, Gilat E. Effects of thyroid hormone on the action potential and membrane currents of guinea pig ventricular myocytes. Pflugers Arch 1987;409:214–216. 3615168 10.1007/BF00584774

[149] Bosch RF , Wang Z, Li GR, Nattel S. Electrophysiological mechanisms by which hypothyroidism delays repolarization in guinea pig hearts. Am J Physiol 1999;277:H211–20. 10409199 10.1152/ajpheart.1999.277.1.H211

[150] Sun ZQ , Ojamaa K, Coetzee WA, Artman M, Klein I. Effects of thyroid hormone on action potential and repolarizing currents in rat ventricular myocytes. Am J Physiol Endocrinol Metab 2000;278:E302–7. 10662715 10.1152/ajpendo.2000.278.2.E302

[151] Hu Y , Jones SV, Dillmann WH. Effects of hyperthyroidism on delayed rectifier K+ currents in left and right murine atria. Am J Physiol Heart Circ Physiol 2005;289:H1448–55. 15894573 10.1152/ajpheart.00828.2004

[152] Rubinstein I , Binah O. Thyroid hormone modulates membrane currents in guinea-pig ventricular myocytes. Naunyn Schmiedebergs Arch Pharmacol 1989;340:705–711. 2615860 10.1007/BF00717748

[153] Han J , Leem C, So I, Kim E, Hong S, Ho W, Sung H, Earm YE. Effects of thyroid hormone on the calcium current and isoprenaline-induced background current in rabbit ventricular myocytes. J Mol Cell Cardiol 1994;26:925–935. 7966361 10.1006/jmcc.1994.1110

[154] Shimoni Y , Banno H. Thyroxine effects on temperature dependence of ionic currents in single rabbit cardiac myocytes. Am J Physiol 1993;265:H1875–83. 8285226 10.1152/ajpheart.1993.265.6.H1875

[155] Steinhilber W , Poensgen J, Rausch U, Kern HF, Scheele GA. Translational control of anionic trypsinogen and amylase synthesis in rat pancreas in response to caerulein stimulation. Proc Natl Acad Sci USA 1988;85:6597–6601. 2457915 10.1073/pnas.85.18.6597 PMC282024

[156] Watanabe H , Ma M, Washizuka T, Komura S, Yoshida T, Hosaka Y, Hatada K, Chinushi M, Yamamoto T, Watanabe K, Aizawa Y. Thyroid hormone regulates mRNA expression and currents of ion channels in rat atrium. Biochem Biophys Res Commun 2003;308:439–444. 12914768 10.1016/s0006-291x(03)01420-7

[157] Sakaguchi Y , Cui G, Sen L. Acute effects of thyroid hormone on inward rectifier potassium channel currents in guinea pig ventricular myocytes. Endocrinology 1996;137:4744–4751. 8895342 10.1210/endo.137.11.8895342

[158] Shimoni Y , Banno H, Clark RB. Hyperthyroidism selectively modified a transient potassium current in rabbit ventricular and atrial myocytes. J Physiol 1992;457:369–389. 1338461 10.1113/jphysiol.1992.sp019383 PMC1175736

[159] Abe A , Yamamoto T, Isome M, Ma M, Yaoita E, Kawasaki K, Kihara I, Aizawa Y. Thyroid hormone regulates expression of shaker-related potassium channel mRNA in rat heart. Biochem Biophys Res Commun 1998;245:226–230. 9535813 10.1006/bbrc.1998.8411

[160] Nishiyama A , Kambe F, Kamiya K, Seo H, Toyama J. Effects of thyroid status on expression of voltage-gated potassium channels in rat left ventricle. Cardiovascular Research 1998;40:343–351. 9893728 10.1016/s0008-6363(98)00135-7

[161] Ma ML , Watanabe K, Watanabe H, Hosaka Y, Komura S, Aizawa Y, Yamamoto T. Different gene expression of potassium channels by thyroid hormone and an antithyroid drug between the atrium and ventricle of rats. Jpn Heart J 2003;44:101–110. 12622442 10.1536/jhj.44.101

[162] Kiss E , Jakab G, Kranias EG, Edes I. Thyroid hormone-induced alterations in phospholamban protein expression. Regulatory effects on sarcoplasmic reticulum Ca2+ transport and myocardial relaxation. Circ Res 1994;75:245–251. 8033338 10.1161/01.res.75.2.245

[163] Chen YC , Chen SA, Chen YJ, Chang MS, Chan P, Lin CI. Effects of thyroid hormone on the arrhythmogenic activity of pulmonary vein cardiomyocytes. J Am Coll Cardiol 2002;39:366–372. 11788233 10.1016/s0735-1097(01)01731-4

[164] Klein LE , Sigel AV, Douglas JA, Eghbali-Webb M. Upregulation of collagen type I gene expression in the ventricular myocardium of thyroidectomized male and female rats. J Mol Cell Cardiol 1996;28:33–42. 8745212 10.1006/jmcc.1996.0004

[165] Yao J , Eghbali M. Decreased collagen gene expression and absence of fibrosis in thyroid hormone-induced myocardial hypertrophy. Response of cardiac fibroblasts to thyroid hormone in vitro. Circ Res 1992;71:831–839. 1381294 10.1161/01.res.71.4.831

[166] Ziegelhoffer-Mihalovicova B , Briest W, Baba HA, Rassler B, Zimmer HG. The expression of mRNA of cytokines and of extracellular matrix proteins in triiodothyronine-treated rat hearts. Mol Cell Biochem 2003;247:61–68. 12841632 10.1023/a:1024153003249

[167] Ghose Roy S , Mishra S, Ghosh G, Bandyopadhyay A. Thyroid hormone induces myocardial matrix degradation by activating matrix metalloproteinase-1. Matrix Biol 2007;26:269–279. 17275272 10.1016/j.matbio.2006.12.005

[168] Nicolini G , Forini F, Kusmic C, Pitto L, Mariani L, Iervasi G. Early and short-term triiodothyronine supplementation prevents adverse postischemic cardiac remodeling: role of transforming growth factor-beta1 and antifibrotic miRNA signaling. Mol Med 2016;21:900–911. 26623926 10.2119/molmed.2015.00140 PMC4818266

[169] Schmidt ED , Cramer SJ, Offringa R. The thyroid hormone receptor interferes with transcriptional activation via the AP-1 complex. Biochem Biophys Res Commun 1993;192:151–160. 8386506 10.1006/bbrc.1993.1394

[170] Lee HW , Klein LE, Raser J, Eghbali-Webb M. An activator protein-1 (AP-1) response element on pro alpha1(l) collagen gene is necessary for thyroid hormone-induced inhibition of promoter activity in cardiac fibroblasts. J Mol Cell Cardiol 1998;30:2495–2506. 9925384 10.1006/jmcc.1998.0811

[171] Kobori H , Ichihara A, Miyashita Y, Hayashi M, Saruta T. Local renin-angiotensin system contributes to hyperthyroidism-induced cardiac hypertrophy. J Endocrinol 1999;160:43–47. 9854175 10.1677/joe.0.1600043 PMC2573048

[172] Hu LW , Benvenuti LA, Liberti EA, Carneiro-Ramos MS, Barreto-Chaves ML. Thyroxine-induced cardiac hypertrophy: influence of adrenergic nervous system versus renin-angiotensin system on myocyte remodeling. Am J Physiol Regul Integr Comp Physiol 2003;285:R1473–80. 12933361 10.1152/ajpregu.00269.2003

[173] Sykora M , Szeiffova BB, Egan BT, Barancik M, Zurmanova J, Rauchova H, Weismann P, Pavelka S, Kurahara LH, Slezak J, Soukup T., Tribulova T. Cardiac Cx43 and ECM responses to altered thyroid status are blunted in spontaneously hypertensive versus normotensive rats. Int J Mol Sci 2019;20: 3758. 10.3390/ijms20153758 PMC6696036 31374823

[174] Weltman NY , Wang D, Redetzke RA, Gerdes AM. Longstanding hyperthyroidism is associated with normal or enhanced intrinsic cardiomyocyte function despite decline in global cardiac function. PLoS One 2012;7:e46655. 23056390 10.1371/journal.pone.0046655 PMC3464244

[175] Hajje G , Saliba Y, Itani T, Moubarak M, Aftimos G, Fares N. Hypothyroidism and its rapid correction alter cardiac remodeling. PLoS One 2014;9:e109753. 25333636 10.1371/journal.pone.0109753 PMC4198123

[176] Irvine SA , Foka P, Rogers SA, Mead JR, Ramji DP. A critical role for the Sp1-binding sites in the transforming growth factor-beta-mediated inhibition of lipoprotein lipase gene expression in macrophages. Nucleic Acids Res 2005;33:1423–1434. 15755745 10.1093/nar/gki280 PMC1062872

[177] Qureshi HY , Sylvester J, El Mabrouk M, Zafarullah M. TGF-beta-induced expression of tissue inhibitor of metalloproteinases-3 gene in chondrocytes is mediated by extracellular signal-regulated kinase pathway and Sp1 transcription factor. J Cell Physiol 2005;203:345–352. 15468069 10.1002/jcp.20228

[178] Zhang X , Yang J, Li Y, Liu Y. Both Sp1 and Smad participate in mediating TGF-beta1-induced HGF receptor expression in renal epithelial cells. Am J Physiol Renal Physiol 2005;288:F16–26. 15339794 10.1152/ajprenal.00318.2003

[179] Chen WJ , Lin KH, Lee YS. Molecular characterization of myocardial fibrosis during hypothyroidism: evidence for negative regulation of the pro-alpha1(I) collagen gene expression by thyroid hormone receptor. Mol Cell Endocrinol 2000;162:45–55. 10854697 10.1016/s0303-7207(00)00203-3

[180] Wu Y , Peng J, Campbell KB, Labeit S, Granzier H. Hypothyroidism leads to increased collagen-based stiffness and re-expression of large cardiac titin isoforms with high compliance. J Mol Cell Cardiol 2007;42:186–195. 17069849 10.1016/j.yjmcc.2006.09.017

[181] Drobnik J , Ciosek J, Slotwinska D, Stempniak B, Zukowska D, Marczynski A, Tosik D, Bartel H, Dabrowski R, Szczepanowska A. Experimental hypothyroidism increases content of collagen and glycosaminoglycans in the heart. J Physiol Pharmacol 2009;60:57–62. 19826182

[182] Zhang Y , Dedkov EI, Teplitsky D, Weltman NY, Pol CJ, Rajagopalan V, Lee B, Gerdes AM. Both hypothyroidism and hyperthyroidism increase atrial fibrillation inducibility in rats. Circ Arrhythm Electrophysiol 2013;6:952–959. 24036190 10.1161/CIRCEP.113.000502 PMC3973490

[183] Zhai T , Cai Z, Zheng J, Ling Y. Impact of hypothyroidism on echocardiographic characteristics of patients with heart valve disease: a single-center propensity score-based study. Front Endocrinol (Lausanne) 2020;11: 554762. 33071970 10.3389/fendo.2020.554762 PMC7542235

[184] Li X , Yang X, Wang Y, Ding L, Wang J, Hua W. The prevalence and prognostic effects of subclinical thyroid dysfunction in dilated cardiomyopathy patients: a single-center cohort study. J Card Fail 2014;20:506–512. 24858054 10.1016/j.cardfail.2014.05.002

[185] Karabag T , Dogan SM, Bayraktaroğlu T, Sayin MR, Buyukuysal C, Akpinar I, Aydin M. Assessment of left atrial mechanical functions in thyroid dysfunction. Pol Arch Med Wewn 2013;123:596–602. 24061556 10.20452/pamw.1970

[186] Ozturk S , Dikbas O, Baltaci D, Ozyasar M, Erdem A, Ayhan SS, Ozlu F, Alcelik A, Tosun M, Yazici M. Evaluation of atrial conduction abnormalities and left atrial mechanical functions in patients with subclinical thyroid disorders. Endokrynol Pol 2012;63:286–293. 22933164

[187] Worku B , Tortolani AJ, Gulkarov I, Isom OW, Klein I. Preoperative hypothyroidism is a risk factor for postoperative atrial fibrillation in cardiac surgical patients. J Card Surg 2015;30:307–312. 25640607 10.1111/jocs.12513

[188] Martinez-Comendador J , Vidal M, Gualis JM, Martin J, Martin CE, Otero E, Castano JM. Subclinical hypothyroidism might increase the risk of postoperative atrial fibrillation after aortic valve replacement. Thorac Cardiovasc Surg 2016;64:427–433. 26121379 10.1055/s-0035-1555753

[189] Jaimes MC , Torrado LAA, Reyes NFS, Mackenzie JC, Mallarino JPU. Hypothyroidism is a risk factor for atrial fibrillation after coronary artery bypass graft. Braz J Cardiovasc Surg 2017;32:475–480. 29267609 10.21470/1678-9741-2017-0080 PMC5731311

[190] Diniz GP , Carneiro-Ramos MS, Barreto-Chaves ML. Angiotensin type 1 (AT1) and type 2 (AT2) receptors mediate the increase in TGF-beta1 in thyroid hormone-induced cardiac hypertrophy. Pflugers Arch 2007;454:75–81. 17206447 10.1007/s00424-006-0192-0

[191] Sanford CF , Griffin EE, Wildenthal K. Synthesis and degradation of myocardial protein during the development and regression of thyroxine-induced cardiac hypertrophy in rats. Circ Res 1978;43:688–694. 152166 10.1161/01.res.43.5.688

[192] Siehl D , Chua BH, Lautensack-Belser N, Morgan HE. Faster protein and ribosome synthesis in thyroxine-induced hypertrophy of rat heart. Am J Physiol 1985;248:C309–19. 3156508 10.1152/ajpcell.1985.248.3.C309

[193] Sossin WS , Fisher JM, Scheller RH. Sorting within the regulated secretory pathway occurs in the trans-Golgi network. J Cell Biol 1990;110:1–12. 2295680 10.1083/jcb.110.1.1 PMC2115992

[194] Kee Z , Kodji X, Brain SD. The role of calcitonin gene related peptide (CGRP) in neurogenic vasodilation and its cardioprotective effects. Front Physiol 2018;9:1249. 30283343 10.3389/fphys.2018.01249 PMC6156372

[195] Karsdal MA , Byrjalsen I, Leeming DJ, Delmas PD, Christiansen C. The effects of oral calcitonin on bone collagen maturation: implications for bone turnover and quality. Osteoporos Int 2008;19:1355–1361. 18385918 10.1007/s00198-008-0603-5

[196] Rodriguez M , Felsenfeld AJ, Torres A, Pederson L, Llach F. Calcitonin, an important factor in the calcemic response to parathyroid hormone in the rat. Kidney Int 1991;40:219–225. 1942770 10.1038/ki.1991.203

[197] Naot D , Musson DS, Cornish J. The activity of peptides of the calcitonin family in bone. Physiol Rev 2019;99:781–805. 30540227 10.1152/physrev.00066.2017

[198] Pondel M. Calcitonin and calcitonin receptors: bone and beyond. Int J Exp Pathol 2000;81:405–422. 11298188 10.1046/j.1365-2613.2000.00176.x PMC2517743

[199] Moreira LM , Paracrine signaling by cardiac calcitonin controls heart fibrogenesis and arrhythmia. *Nature* 2020. 10.1038/s41586-020-2890-8 33149301

[200] Masi L , Brandi ML. Calcitonin and calcitonin receptors. Clin Cases Miner Bone Metab 2007;4:117–122. 22461211 PMC2781237

[201] Stroop SD , Thompson DL, Kuestner RE, Moore EE. A recombinant human calcitonin receptor functions as an extracellular calcium sensor. J Biol Chem 1993;268:19927–19930. 8397191

[202] Chen Y , Shyu JF, Santhanagopal A, Inoue D, David JP, Dixon SJ, Horne WC, Baron R. The calcitonin receptor stimulates Shc tyrosine phosphorylation and Erk1/2 activation. Involvement of Gi, protein kinase C, and calcium. J Biol Chem 1998;273:19809–19816. 9677414 10.1074/jbc.273.31.19809

[203] Lin HY , Harris TL, Flannery MS, Aruffo A, Kaji EH, Gorn A, Kolakowski LF Jr., Yamin M, Lodish HF, Goldring SR. Expression cloning and characterization of a porcine renal calcitonin receptor. Trans Assoc Am Physicians 1991;104:265–272. 1668988

[204] Nicholson GC , Moseley JM, Sexton PM, Mendelsohn FA, Martin TJ. Abundant calcitonin receptors in isolated rat osteoclasts. Biochemical and autoradiographic characterization. J Clin Invest 1986;78:355–360. 3016026 10.1172/JCI112584 PMC423551

[205] Albrandt K , Brady EM, Moore CX, Mull E, Sierzega ME, Beaumont K. Molecular cloning and functional expression of a third isoform of the human calcitonin receptor and partial characterization of the calcitonin receptor gene. Endocrinology 1995;136:5377–5384. 7588285 10.1210/endo.136.12.7588285

[206] Basuyau JP , Mallet E, Leroy M, Brunelle P. Reference intervals for serum calcitonin in men, women, and children. Clin Chem 2004;50:1828–1830. 15388660 10.1373/clinchem.2003.026963

[207] Locke AE , Kahali B, Berndt SI, Justice AE, Pers TH, Day FR, Powell C, Vedantam S, Buchkovich ML, Yang J, Croteau-Chonka DC, Esko T, Fall T, Ferreira T, Gustafsson S, Kutalik Z, Luan J, Mägi R, Randall JC, Winkler TW, Wood AR, Workalemahu T, Faul JD, Smith JA, Hua Zhao J, Zhao W, Chen J, Fehrmann R, Hedman ÅK, Karjalainen J, Schmidt EM, Absher D, Amin N, Anderson D, Beekman M, Bolton JL, Bragg-Gresham JL, Buyske S, Demirkan A, Deng G, Ehret GB, Feenstra B, Feitosa MF, Fischer K, Goel A, Gong J, Jackson AU, Kanoni S, Kleber ME, Kristiansson K, Lim U, Lotay V, Mangino M, Mateo Leach I, Medina-Gomez C, Medland SE, Nalls MA, Palmer CD, Pasko D, Pechlivanis S, Peters MJ, Prokopenko I, Shungin D, Stančáková A, Strawbridge RJ, Ju Sung Y, Tanaka T, Teumer A, Trompet S, van der Laan SW, van Setten J, Van Vliet-Ostaptchouk JV, Wang Z, Yengo L, Zhang W, Isaacs A, Albrecht E, Ärnlöv J, Arscott GM, Attwood AP, Bandinelli S, Barrett A, Bas IN, Bellis C, Bennett AJ, Berne C, Blagieva R, Blüher M, Böhringer S, Bonnycastle LL, Böttcher Y, Boyd HA, Bruinenberg M, Caspersen IH, Ida Chen Y-D, Clarke R, Warwick Daw E, de Craen AJM, Delgado G, Dimitriou M, Doney ASF, Eklund N, Estrada K, Eury E, Folkersen L, Fraser RM, Garcia ME, Geller F, Giedraitis V, Gigante B, Go AS, Golay A, Goodall AH, Gordon SD, Gorski M, Grabe H-J, Grallert H, Grammer TB, Gräßler J, Grönberg H, Groves CJ, Gusto G, Haessler J, Hall P, Haller T, Hallmans G, Hartman CA, Hassinen M, Hayward C, Heard-Costa NL, Helmer Q, Hengstenberg C, Holmen O, Hottenga J-J, James AL, Jeff JM, Johansson Å, Jolley J, Juliusdottir T, Kinnunen L, Koenig W, Koskenvuo M, Kratzer W, Laitinen J, Lamina C, Leander K, Lee NR, Lichtner P, Lind L, Lindström J, Sin Lo K, Lobbens S, Lorbeer R, Lu Y, Mach F, Magnusson PKE, Mahajan A, McArdle WL, McLachlan S, Menni C, Merger S, Mihailov E, Milani L, Moayyeri A, Monda KL, Morken MA, Mulas A, Müller G, Müller-Nurasyid M, Musk AW, Nagaraja R, Nöthen MM, Nolte IM, Pilz S, Rayner NW, Renstrom F, Rettig R, Ried JS, Ripke S, Robertson NR, Rose LM, Sanna S, Scharnagl H, Scholtens S, Schumacher FR, Scott WR, Seufferlein T, Shi J, Vernon Smith A, Smolonska J, Stanton AV, Steinthorsdottir V, Stirrups K, Stringham HM, Sundström J, Swertz MA, Swift AJ, Syvänen A-C, Tan S-T, Tayo BO, Thorand B, Thorleifsson G, Tyrer JP, Uh H-W, Vandenput L, Verhulst FC, Vermeulen SH, Verweij N, Vonk JM, Waite LL, Warren HR, Waterworth D, Weedon MN, Wilkens LR, Willenborg C, Wilsgaard T, Wojczynski MK, Wong A, Wright AF, Zhang Q, Brennan EP, Choi M, Dastani Z, Drong AW, Eriksson P, Franco-Cereceda A, Gådin JR, Gharavi AG, Goddard ME, Handsaker RE, Huang J, Karpe F, Kathiresan S, Keildson S, Kiryluk K, Kubo M, Lee J-Y, Liang L, Lifton RP, Ma B, McCarroll SA, McKnight AJ, Min JL, Moffatt MF, Montgomery GW, Murabito JM, Nicholson G, Nyholt DR, Okada Y, Perry JRB, Dorajoo R, Reinmaa E, Salem RM, Sandholm N, Scott RA, Stolk L, Takahashi A, Tanaka T, van’t Hooft FM, Vinkhuyzen AAE, Westra H-J, Zheng W, Zondervan KT, Heath AC, Arveiler D, Bakker SJL, Beilby J, Bergman RN, Blangero J, Bovet P, Campbell H, Caulfield MJ, Cesana G, Chakravarti A, Chasman DI, Chines PS, Collins FS, Crawford DC, Adrienne Cupples L, Cusi D, Danesh J, de Faire U, den Ruijter HM, Dominiczak AF, Erbel R, Erdmann J, Eriksson JG, Farrall M, Felix SB, Ferrannini E, Ferrières J, Ford I, Forouhi NG, Forrester T, Franco OH, Gansevoort RT, Gejman PV, Gieger C, Gottesman O, Gudnason V, Gyllensten U, Hall AS, Harris TB, Hattersley AT, Hicks AA, Hindorff LA, Hingorani AD, Hofman A, Homuth G, Kees Hovingh G, Humphries SE, Hunt SC, Hyppönen E, Illig T, Jacobs KB, Jarvelin M-R, Jöckel K-H, Johansen B, Jousilahti P, Wouter Jukema J, Jula AM, Kaprio J, Kastelein JJP, Keinanen-Kiukaanniemi SM, Kiemeney LA, Knekt P, Kooner JS, Kooperberg C, Kovacs P, Kraja AT, Kumari M, Kuusisto J, Lakka TA, Langenberg C, Le Marchand L, Lehtimäki T, Lyssenko V, Männistö S, Marette A, Matise TC, McKenzie CA, McKnight B, Moll FL, Morris AD, Morris AP, Murray JC, Nelis M, Ohlsson C, Oldehinkel AJ, Ong KK, Madden PAF, Pasterkamp G, Peden JF, Peters A, Postma DS, Pramstaller PP, Price JF, Qi L, Raitakari OT, Rankinen T, Rao DC, Rice TK, Ridker PM, Rioux JD, Ritchie MD, Rudan I, Salomaa V, Samani NJ, Saramies J, Sarzynski MA, Schunkert H, Schwarz PEH, Sever P, Shuldiner AR, Sinisalo J, Stolk RP, Strauch K, Tönjes A, Trégouët D-A, Tremblay A, Tremoli E, Virtamo J, Vohl M-C, Völker U, Waeber G, Willemsen G, Witteman JC, Carola Zillikens M, Adair LS, Amouyel P, Asselbergs FW, Assimes TL, Bochud M, Boehm BO, Boerwinkle E, Bornstein SR, Bottinger EP, Bouchard C, Cauchi S, Chambers JC, Chanock SJ, Cooper RS, de Bakker PIW, Dedoussis G, Ferrucci L, Franks PW, Froguel P, Groop LC, Haiman CA, Hamsten A, Hui J, Hunter DJ, Hveem K, Kaplan RC, Kivimaki M, Kuh D, Laakso M, Liu Y, Martin NG, März W, Melbye M, Metspalu A, Moebus S, Munroe PB, Njølstad I, Oostra BA, Palmer CNA, Pedersen NL, Perola M, Pérusse L, Peters U, Power C, Quertermous T, Rauramaa R, Rivadeneira F, Saaristo TE, Saleheen D, Sattar N, Schadt EE, Schlessinger D, Eline Slagboom P, Snieder H, Spector TD, Thorsteinsdottir U, Stumvoll M, Tuomilehto J, Uitterlinden AG, Uusitupa M, van der Harst P, Walker M, Wallaschofski H, Wareham NJ, Watkins H, Weir DR, Wichmann H-E, Wilson JF, Zanen P, Borecki IB, Deloukas P, Fox CS, Heid IM, O’Connell JR, Strachan DP, Stefansson K, van Duijn CM, Abecasis GR, Franke L, Frayling TM, McCarthy MI, Visscher PM, Scherag A, Willer CJ, Boehnke M, Mohlke KL, Lindgren CM, Beckmann JS, Barroso I, North KE, Ingelsson E, Hirschhorn JN, Loos RJF, Speliotes EK, The LifeLines Cohort Study. Genetic studies of body mass index yield new insights for obesity biology. Nature 2015;518:197–206. 25673413 10.1038/nature14177 PMC4382211

[208] Chugh SS , Havmoeller R, Narayanan K, Singh D, Rienstra M, Benjamin EJ, Gillum RF, Kim YH, McAnulty JH Jr, Zheng ZJ, Forouzanfar MH, Naghavi M, Mensah GA, Ezzati M, Murray CJ. Worldwide epidemiology of atrial fibrillation: a Global Burden of Disease 2010 Study. Circulation 2014;129:837–847. 24345399 10.1161/CIRCULATIONAHA.113.005119 PMC4151302

[209] Heijman J , Guichard JB, Dobrev D, Nattel S. Translational challenges in atrial fibrillation. Circ Res 2018;122:752–773. 29496798 10.1161/CIRCRESAHA.117.311081

[210] Cuparencu B , Ticsa I, Barzu T, Gozariu SVL. Effect of calcitonin on certain experimental models of arrhythmia. Therapie 1975;555–563. 1209530

[211] Chiba S , Himori N. Effects of salmon calcitonin on SA nodal pacemaker activity and contractility in isolated, blood-perfused atrial and papillary muscle preparations of dogs. Jpn Heart J 1977;18:214–220. 870729 10.1536/ihj.18.214

[212] Hagenacker T , Ledwig D, Busselberg D. Additive inhibitory effects of calcitonin and capsaicin on voltage activated calcium channel currents in nociceptive neurones of rat. Brain Res Bull 2011;85:75–80. 21335070 10.1016/j.brainresbull.2011.02.006

[213] Zuo Q , Claveau D, Hilal G, Leclerc M, Brunette MG. Effect of calcitonin on calcium transport by the luminal and basolateral membranes of the rabbit nephron. Kidney Int 1997;51:1991–1999. 9186893 10.1038/ki.1997.271

[214] Yamashita N , Hagiwara S. Membrane depolarization and intracellular Ca2+ increase caused by high external Ca2+ in a rat calcitonin-secreting cell line. J Physiol 1990;431:243–267. 1712840 10.1113/jphysiol.1990.sp018329 PMC1181773

[215] Sand O , Jonsson L, Nielsen M, Holm R, Gautvik KM. Electrophysiological properties of calcitonin-secreting cells derived from human medullary thyroid carcinoma. Acta Physiol Scand 1986;126:173–179. 3705981 10.1111/j.1748-1716.1986.tb07803.x

[216] Xie W , Santulli G, Reiken SR, Yuan Q, Osborne BW, Chen BX, Marks AR. Mitochondrial oxidative stress promotes atrial fibrillation. Sci Rep 2015;5:11427. 26169582 10.1038/srep11427 PMC4501003

[217] Borle AB. Regulation of cellular calcium metabolism and calcium transport by calcitonin. J Membr Biol 1975;21:125–146. 172633 10.1007/BF01941066

[218] Ito A , Takeda M, Yoshimura T, Komatsu T, Ohno T, Kuriyama H, Matsuda A, Yoshimura M. Anti-hyperalgesic effects of calcitonin on neuropathic pain interacting with its peripheral receptors. Mol Pain 2012;8:42. 22676202 10.1186/1744-8069-8-42 PMC3517395

[219] El Hajjaji H , Williams JM, Devogelaer JP, Lenz ME, Thonar EJ, Manicourt DH. Treatment with calcitonin prevents the net loss of collagen, hyaluronan and proteoglycan aggregates from cartilage in the early stages of canine experimental osteoarthritis. Osteoarthritis Cartilage 2004;12:904–911. 15501406 10.1016/j.joca.2004.08.005

[220] Yamaguchi M , Watanabe Y, Ohtani T, Uezumi A, Mikami N, Nakamura M, Sato T, Ikawa M, Hoshino M, Tsuchida K, Miyagoe-Suzuki Y, Tsujikawa K, Takeda S, Yamamoto H, Fukada S. Calcitonin receptor signaling inhibits muscle stem cells from escaping the quiescent state and the niche. Cell Rep 2015;13:302–314. 26440893 10.1016/j.celrep.2015.08.083

[221] Zelniker TA , Bonaca MP, Furtado RHM, Mosenzon O, Kuder JF, Murphy SA, Bhatt DL, Leiter LA, McGuire DK, Wilding JPH, Budaj A, Kiss RG, Padilla F, Gause-Nilsson I, Langkilde AM, Raz I, Sabatine MS, Wiviott SD. Effect of dapagliflozin on atrial fibrillation in patients with type 2 diabetes mellitus: insights from the DECLARE-TIMI 58 trial. Circulation 2020;141:1227–1234. 31983236 10.1161/CIRCULATIONAHA.119.044183

[222] Monami M , Nreu B, Scatena A, Giannini S, Andreozzi F, Sesti G, Mannucci E. Glucagon-like peptide-1 receptor agonists and atrial fibrillation: a systematic review and meta-analysis of randomised controlled trials. J Endocrinol Invest 2017;40:1251–1258. 28569363 10.1007/s40618-017-0698-7

[223] Nreu B , Dicembrini I, Tinti F, Sesti G, Mannucci E, Monami M. Major cardiovascular events, heart failure, and atrial fibrillation in patients treated with glucagon-like peptide-1 receptor agonists: an updated meta-analysis of randomized controlled trials. Nutr Metab Cardiovasc Dis 2020;30:1106–1114. 32448716 10.1016/j.numecd.2020.03.013

[224] Priest C , Tontonoz P. Inter-organ cross-talk in metabolic syndrome. Nat Metab 2019;1:1177–1188. 32694672 10.1038/s42255-019-0145-5

[225] Iskandar S , Reddy M, Afzal MR, Rajasingh J, Atoui M, Lavu M, Atkins D, Bommana S, Umbarger L, Jaeger M, Pimentel R, Dendi R, Emert M, Turagam M, Di BL, Lakkireddy NA. D. Use of oral steroid and its effects on atrial fibrillation recurrence and inflammatory cytokines post ablation - the steroid AF study. J Atr Fibrillation 2017;9: 1604. 29250282 10.4022/jafib.1604 PMC5673398

[226] Al-Shawabkeh Z , Al-Nawaesah K, Anzeh RA, Al-Odwan H, Al-Rawashdeh WA, Altaani H. Use of short-term steroids in the prophylaxis of atrial fibrillation after cardiac surgery. J Saudi Heart Assoc 2017;29:23–29. 28127215 10.1016/j.jsha.2016.03.005 PMC5247295

[227] Hsieh YC , Hung CY, Li CH, Liao YC, Huang JL, Lin CH, Wu TJ. Angiotensin-receptor blocker, angiotensin-converting enzyme inhibitor, and risks of atrial fibrillation: a nationwide cohort study. Medicine 2016;95:e3721. 27196491 10.1097/MD.0000000000003721 PMC4902433

[228] Alexandre J , Dolladille C, Douesnel L, Font J, Dabrowski R, Shavit L, Legallois D, Funck-Brentano C, Champ-Rigot L, Ollitrault P, Beygui F, Bejan-Angoulvant T, Parienti JJ, Milliez P. Effects of mineralocorticoid receptor antagonists on atrial fibrillation occurrence: a systematic review, meta-analysis, and meta-regression to identify modifying factors. J Am Heart Assoc 2019;8:e013267. 31711383 10.1161/JAHA.119.013267 PMC6915291

[229] Oni OA , Sharma R, Chen G, Sharma M, Gupta K, Dawn B, Sharma R, Parashara D, Savin VJ, Cherian G, Ambrose JA, Barua RS. Normalization of testosterone levels after testosterone replacement therapy is not associated with reduced myocardial infarction in smokers. Mayo Clin Proc Innov Qual Outcomes 2017;1:57–66. 30225402 10.1016/j.mayocpiqo.2017.05.003 PMC6135014

[230] Bretler DM , Hansen PR, Lindhardsen J, Ahlehoff O, Andersson C, Jensen TB, Raunso J, Torp-Pedersen C, Gislason GH. Hormone replacement therapy and risk of new-onset atrial fibrillation after myocardial infarction – a nationwide cohort study. PLoS One 2012;7:e51580. 23284717 10.1371/journal.pone.0051580 PMC3524193

[231] Pham TV , Robinson RB, Danilo P Jr, Rosen MR. Effects of gonadal steroids on gender-related differences in transmural dispersion of L-type calcium current. Cardiovascular Research 2002;53:752–762. 11861045 10.1016/s0008-6363(01)00449-7

[232] Linde C , Bongiorni MG, Birgersdotter-Green U, Curtis AB, Deisenhofer I, Furokawa T, Gillis AM, Haugaa KH, Lip GYH, Van Gelder I, Malik M, Poole J, Potpara T, Savelieva I, Sarkozy A. ESC Scientific Document Group. Sex differences in cardiac arrhythmia: a consensus document of the European Heart Rhythm Association, endorsed by the Heart Rhythm Society and Asia Pacific Heart Rhythm Society. Europace 2018;20:1565–1565. 29961863 10.1093/europace/euy067

[233] Rajagopalan V , Zhang Y, Ojamaa K, Chen YF, Pingitore A, Pol CJ, Saunders D, Balasubramanian K, Towner RA, Gerdes AM. Safe oral triiodo-L-thyronine therapy protects from post-infarct cardiac dysfunction and arrhythmias without cardiovascular adverse effects. PLoS One 2016;11:e0151413. 26981865 10.1371/journal.pone.0151413 PMC4794221

[234] Brenta G , Danzi S, Klein I. Potential therapeutic applications of thyroid hormone analogs. Nat Clin Pract Endocrinol Metab 2007;3:632–640. 17710084 10.1038/ncpendmet0590

[235] Singer FR , Aldred JP, Neer RM, Krane SM, Potts JT Jr, Bloch KJ. An evaluation of antibodies and clinical resistance to salmon calcitonin. J Clin Invest 1972;51:2331–2338. 4674133 10.1172/JCI107044 PMC292399

[236] Andrade JG , Aguilar M, Atzema C, Bell A, Cairns JA, Cheung CC, Cox JL, Dorian P, Gladstone DJ, Healey JS, Khairy P, Leblanc K, McMurtry MS, Mitchell LB, Nair GM, Nattel S, Parkash R, Pilote L, Sandhu RK, Sarrazin J-F, Sharma M, Skanes AC, Talajic M, Tsang TSM, Verma A, Verma S, Whitlock R, Wyse DG, Macle L. Members of the Secondary Panel. The 2020 Canadian Cardiovascular Society/Canadian Heart Rhythm Society Comprehensive Guidelines for the Management of Atrial Fibrillation. Can J Cardiol 2020;36:1847–1948. 33191198 10.1016/j.cjca.2020.09.001

